# Targeted sonogenetic modulation of GABAergic interneurons in the hippocampal CA1 region in status epilepticus

**DOI:** 10.7150/thno.96598

**Published:** 2024-10-07

**Authors:** Tao Xu, Dandan Tan, You Wang, Chen Gong, Jinxian Yuan, Xiaolan Yang, Yuetao Wen, Yuenan Ban, Minxue Liang, Yaqin Hu, Yang Cao, Yangmei Chen, Haitao Ran

**Affiliations:** 1 Department of Neurology, the Second Affiliated Hospital of Chongqing Medical University, Chongqing 400010, China.; 2 Department of Ultrasound, Chongqing Key Laboratory of Ultrasound Molecular Imaging, the Second Affiliated Hospital of Chongqing Medical University, Chongqing 400010, China.

**Keywords:** sonogenetic, status epilepticus, hippocampus, parvalbumin interneurons, somatostatin interneurons

## Abstract

**Rationale:** Sonogenetics is an advanced ultrasound-based neurostimulation approach for targeting neurons in specific brain regions. However, the role of sonogenetics in treating status epilepticus (SE) remains unclear. Here, we aimed to investigate the effects of ultrasound neurostimulation and MscL-G22S (a mechanosensitive ion channel that mediates Ca^2+^ influx)-mediated sonogenetics (MG-SOG) in a mouse model of kainic acid (KA)-induced SE.

**Methods:** For MG-SOG, a Cre-dependent AAV expressing MscL-G22S was injected into parvalbumin (PV)-cre and somatostatin (SST)-cre mice to induce the expression of MscL-G22S-EGFP in PV interneurons (PV-INs) and SST interneurons (SST-INs), respectively; mice were stimulated with continuous pulses of ultrasound stimulation during the latency of generalized seizures (GSs), the latency to SE, in SE model mice**.** We performed calcium fiber photometry, patch-clamp recording, local field potential recording, and SE monitoring to investigate the role of MG-SOG in treating SE.

**Results:** First, we observed obvious neuronal activation in the hippocampal CA1 region in SE model mice. Both excitatory neurons (ENs) and GABAergic interneurons (GABA-INs) in the CA1 region were activated in SE model mice; however, the inhibitory effect of GABA-INs on ENs seemed to be insufficient to reduce EN excitability despite the increased activation of GABA-INs in SE model mice. Thus, we speculated that MG-SOG-induced activation of GABA-INs, mainly SST-INs and PV-INs, in the CA1 region may protect against SE. We found that MG-SOG-mediated PV-IN activation in the CA1 region ameliorated SE and changed SE-related electrophysiological abnormalities in the CA1 region; however, MG-SOG-induced SST-IN activation in the CA1 region did not ameliorate SE.

**Conclusions:** MG-SOG-mediated activation of PV-INs had a positive effect on relieving SE. Our work may promote the development of sonogenetic neurostimulation techniques for treating SE.

## Introduction

Status epilepticus (SE) is a common and life-threatening neurological emergency that usually requires urgent management and treatment 1, 2. SE is defined as prolonged seizure episodes or multiple seizures (without termination or recovery to baseline) persisting for more than 5 min due to the failure of seizure cessation or abnormal seizure initiation involving the dysfunction of excitatory neurons (ENs) or GABAergic interneurons (GABA-INs), which causes excitation-inhibition imbalance (EI-IM) in the brain 1, 3-5. Epidemiological data indicate that SE is one of the main causes of premature death in people with epilepsy 6, 7. Moreover, SE can lead to a series of adverse effects, including neuronal injury, neuronal death, and abnormal neural network generation, further leading to long-term or irreversible neurological damage 8, 9.

Current guidelines for SE treatment recommend three levels of treatment: benzodiazepines (BZPs), second-line therapy with antiseizure medications (ASMs) and third-line treatment with an anesthetic 1, 10. However, approximately one-fifth of SE patients have poor responses to first-line BZPs and second-line ASMs and develop refractory status epilepticus (RSE) 11-13. Notably, in some cases, RSE may persist for or recur within 24 hours after the onset of anesthetic treatment or may recur following the withdrawal of the anesthetic and ultimately progress to super refractory status epilepticus, which is associated with increased in-hospital mortality 14, 15. Thus, it is necessary to explore novel therapeutic methods for treating SE.

Recently, ultrasound neurostimulation, which mainly utilizes low-intensity focused ultrasound (LIFU) to produce mild mechanical forces with low energy rather than high-energy ultrasound, which can cause thermal ablation of brain tissue, has been shown to have great potential for regulating brain functions and treating central nervous system (CNS) diseases, including epilepsy, essential tremor, Alzheimer's disease, and Parkinson's disease, in humans and animals 16-18. Thus, ultrasound neurostimulation is considered a promising candidate for treating CNS diseases and has attracted increasing amounts of attention from researchers 19. During ultrasound neurostimulation, the ultrasound frequency is negatively associated with the ability of the ultrasound waves to penetrate the skull or brain tissue but is positively associated with the focused size of the ultrasound field 20. Therefore, LIFU waves with a low frequency (less than 1 MHz) can penetrate brain tissue and reach deep brain regions more effectively than those with a high frequency but have poorer spatial specificity 20, 21. Thus, ultrasound neurostimulation is limited by insufficient spatial specificity or cell-type specificity within the brain.

Sonogenetics is an advanced ultrasound-based neurostimulation approach that can be combined with the overexpression of mechanosensitive ion channels in specific cells 22, 23. Mechanosensitive ion channels, which are overexpressed in specific neurons *in vivo* or *in vitro*, can enhance the response of target neurons to ultrasound stimulation, thereby increasing the cell-type specificity of ultrasound stimulation 22, 23. Therefore, sonogenetics can increase the spatial specificity and cell type specificity of LIFU when mechanosensitive ion channels are overexpressed, increasing the effectiveness of LIFU in stimulating the brain 22, 24. Recently, sonogenetics has been proven to activate specific neurons in brain regions to regulate neural circuit functions and control limb movements in animal models 22, 24. MscL-G22S, a well-established large-conductance mechanosensitive ion channel that mediates Ca^2+^ influx, is able to convert mild mechanical stimulation into effective and rapid cellular activation 22, 24. *In vivo* and *in vitro*, neurons expressing MscL-G22S show significantly greater Ca^2+^ influx upon mild ultrasound stimulation, which causes neuronal activation 22, 24. In the absence of ultrasound stimulation, MscL-G22S expressed in neurons has little influence on Ca^2+^ influx and neuronal activation; therefore, MscL-G22S is a promising candidate mechanosensitive ion channel target for sonogenetics-based neurostimulation 22, 24.

To date, the effectiveness of several neuromodulatory approaches for treating chronic seizures in epilepsy patients has been preliminarily evaluated in clinical and animal studies 17, 25. However, the effectiveness of neuromodulatory approaches, especially ultrasound neurostimulation, in treating SE has rarely been evaluated, and the effect of ultrasound neurostimulation in treating SE has not been fully elucidated. Given the abovementioned advantages of sonogenetics in regulating brain function, in the present study, we aimed to investigate the potential effects of ultrasound neurostimulation and MscL-G22S-mediated sonogenetics (MG-SOG) in alleviating SE via the activation of GABAergic neurons in the hippocampal CA1 region in a mouse model of kainic acid (KA)-induced SE.

## Results

### Changes in neuronal activation in the hippocampal CA1 region of SE model mice

**Figure 1:**
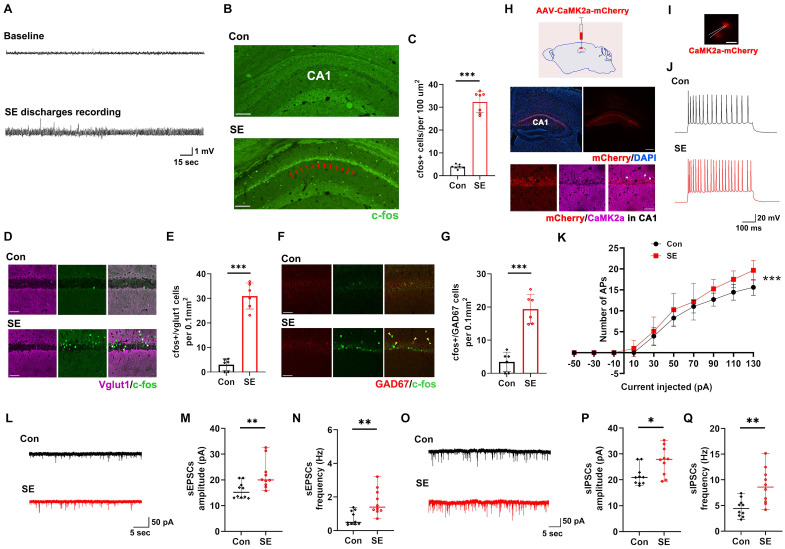
The expression of c-fos and the electrophysiological properties of ENs in the hippocampal CA1 region of KA-induced SE model mice. **(A)** Representative SE discharges from LFP recordings of the hippocampal CA1 region. **(B)** Representative images of c-fos expression in hippocampal CA1 region of mice in the Con group and SE group; the red arrows indicate obvious c-fos expression in the CA1 region in the SE group; *scale bar*, 100 μm. **(C)** Comparisons of c-fos expression levels in hippocampal CA1 region between mice in the Con group and the SE group (n = 7).** (D)** Representative images of c-fos and Vglut1 colocalization in the hippocampal CA1 region in the Con group and SE group; the white arrows indicate the colocalization of Vglut1 and c-fos; *scale bar*, 50 μm. **(E)** Comparison of the number of cells expressing both c-fos and Vglut1 in the CA1 region between the Con group and the SE group (n = 6). **(F)** Representative images of c-fos and GAD67 colocalization in the hippocampal CA1 region in the Con group and SE group; the yellow arrows indicate the colocalization of GAD67 and c-fos;* scale bar*, 50 μm. **(G)** Comparison of the number of cells expressing both c-fos and GAD67 in the CA1 region between the Con group and the SE group (n = 6). **(H)** Schematic diagram of stereotactic injection of AAV-CaMK2a-mCherry into the CA1 region to label ENs; representative fluorescence image of mCherry in the hippocampus (*scale bar*, 400 μm) and the colocalization of CaMK2a and mCherry in the CA1 region; the white arrows indicate the colocalization of CaMK2a and mCherry (*scale bar*, 50 μm). **(I)** Representative fluorescence image of CaMK2a+ ENs expressing mCherry (red) during patch clamp recording (*scale bar*, 20 μm) and **(J)** representative traces of APs produced by ENs from the Con group and SE group. **(K)** Comparison of the EN AP number versus injected current curve between the Con group and SE group (n = 12 cells per group from 4 mice). **(L)** Representative traces of sEPSCs in ENs in the Con group and SE group. Comparisons of **(M)** the amplitude and** (N)** frequency of sEPSCs in ENs between the Con group and the SE group (n = 10 cells per group from 4 mice). **(O)** Representative traces of sIPSCs in ENs in the Con group and SE group. Comparisons of **(P)** the amplitude and** (Q)** frequency of sIPSCs in ENs between the Con group and the SE group (n = 10 cells per group from 4 mice). Student's *t* test in C, E, G; the data are the mean ± SD. Two-way RM-ANOVA followed by Bonferroni *post hoc* test in K; the data are the mean ± SD. The Mann‒Whitney test in M, N, P, Q; the data are the median and range; *P < 0.05, **P < 0.01, and ***P < 0.001.

First, we aimed to identify the brain region closely associated with SE in a KA-induced SE mouse model. Previous studies have indicated that the EI-IM in the hippocampal CA1 region is associated with SE 26-28. Thus, we speculated that the hippocampal CA1 region plays a key role in SE. First, we monitored SE discharges by recording local field potentials (LFPs) in the hippocampus **(Figure 1A)**. Next, we investigated the expression of the neuronal activation marker c-fos in the hippocampal CA1 region of SE model mice and detected a greater level of c-fos expression in the hippocampal CA1 region in the SE group than in the control group **(Figure 1B-C)**. Based on these data, we speculated that the hippocampal CA1 region plays a key role in regulating the generation and progression of SE. In the hippocampal CA1 region, Vglut1-positive neurons (indicating glutaminergic neurons) **(Figure 1D-E)** and glutamate decarboxylase (GAD)-67-positive neurons (indicating GABA-INs) **(Figure 1F-G)** exhibited higher c-fos expression in the SE group than in the control group, indicating that both ENs and GABA-INs showed obviously increased activation in the SE group.

Furthermore, we performed patch clamp recording to investigate the electrophysiological properties of ENs in the CA1 region in the SE group and control group. To label ENs in the CA1 region, we injected an adeno-associated virus (AAV) vector expressing mCherry under the control of the Ca^2+^/calmodulin-dependent protein kinase 2α (CaMK2α) promoter (AAV-CaMK2a-mCherry) into the CA1 region to induce the expression of mCherry in ENs **(Figure 1H)**. Subsequent patch clamp recordings revealed that action potential (AP) firing by CaMK2a-mCherry-positive neurons (indicating ENs) in the CA1 region increased in the SE group compared with the control group as the injected current increased **(Figure 1I-K)**, suggesting increased intrinsic excitability of ENs in the SE group. Subsequent analyses of the intrinsic physiological properties of the APs revealed that the AP half-width was smaller in the SE group than in the control group **(Table S1)**. There were no significant differences in peak amplitude, resting membrane potential (RMP), input resistance, membrane capacitance, or threshold potential **(Table S1)** between the SE group and the control group. Furthermore, we investigated the synaptic transmission of ENs in the CA1 region and found that the amplitude and frequency of spontaneous excitatory postsynaptic currents (sEPSCs) were significantly greater in the SE group than in the control group **(Figure 1L-N)**, indicating increased excitatory synaptic transmission (EST) by ENs in the SE group. The inhibitory effect of GABA-INs on ENs via inhibitory synaptic transmission (IST) plays a key role in regulating EN excitability 29, 30. Thus, we investigated the effects of IST on ENs and reported that the amplitude and frequency of spontaneous inhibitory postsynaptic currents (sIPSCs) were greater in the SE group than in the control group **(Figure 1O-Q)**.

Taken together, these findings showed that activation of the hippocampal CA1 region was significantly elevated in SE model mice and that both ENs and GABA-INs were activated in these mice. In the SE group, although the ENs in the CA1 region were hyperexcitable and received increased EST and IST, EST to these neurons was more significantly increased than IST to these neurons. Based on these findings, we speculated that the hippocampal CA1 region plays a key role in SE. Although GABA-INs inhibited ENs via increased activation of GABA-INs in SE model mice, this inhibitory effect was insufficient to reduce EN excitability; therefore, activating GABA-INs in the CA1 region may enhance GABA-IN-mediated inhibition of ENs, further reducing EN excitability and ultimately alleviating SE.

### Parameters used for ultrasound stimulation and effects of ultrasound stimulation on neuronal activation in the CA1 region

** Neuronal activity in the hippocampal CA1 region upon LIFU stimulation.** The right side of the mouse brain was stimulated at an acoustic pressure of 0.38 MPa (the stimulation parameters are shown in **Figure S1**). To measure the response of neurons to LIFU stimulation (LIFU-stim), we first induced the expression of the ultrasensitive calcium indicator GCaMp6s 31 in neurons in the right hippocampal CA1 region via stereotactic injection of AAV-hSyn-GCaMp6s into this brain area; subsequently, a photometric fiber with a ceramic ferrule implanted into the CA1 region was used to measure the response of neurons to LIFU stimulation **(Figure S2A-B)**. We first used a single pulse of LIFU-stim (1 second stimulation duration; 650 kHz central frequency) **(Figure S2C)** and found that this single pulse promoted neuronal activation in the right hippocampal CA1 region for approximately 15 s **(Figure S2D)**. Furthermore, upon application of continuous pulses of LIFU stimulation with an interval of 15 s between pulses **(Figure S2E)**, CA1 neurons were stably activated **(Figure S2F)**.

** EN and GABA-IN activities in the hippocampal CA1 region upon LIFU stimulation.** Next, we used fiber photometry to measure the effects of LIFU stimulation on the Ca^2+^ responses of ENs and GABA-INs. The results indicated that the Ca^2+^ responses of both ENs and GABA-INs were stronger in the LIFU-stim group than in the sham group; moreover, in the LIFU-stim group, the increase in the Ca^2+^ response of ENs appeared to be more obvious than the increase in GABA-INs **(Figure S3)**. These data suggested that LIFU stimulation was able to activate both ENs and GABA-INs in the CA1 region but preferentially activated ENs.

**The effect of LIFU stimulation on LFPs in the hippocampal CA1 region.** LFP recordings of fast gamma oscillations (FGRs) (90-150 Hz) and ripple oscillations (ROs) (110-200 Hz) in the hippocampal CA1 region **(Figure S4)** reflect AP generation and synchronization in ENs, respectively, both of which represent the excitability of the local neuronal network (LNN) in the CA1 region 32. Thus, FGR and RO recordings were used to determine the effect of LIFU stimulation on the excitability of the LNN in the CA1 region **(Figure S5A)**. The sums of the spectra of both FGRs and ROs in the CA1 region were significantly increased upon single-pulse LIFU stimulation **(Figure S5B-D)**, indicating that LIFU stimulation effectively promoted the activation of LNNs in the CA1 region. These data demonstrated that the stimulating effect of LIFU could reach the hippocampal CA1 region, affecting the LNN in this region.

**The effect of LIFU stimulation on temperature variation in the hippocampal CA1 region.** Prolonged LIFU stimulation, even at low energies, may produce mild thermal effects, which are defined as extracellular temperature variations equal to or greater than one degree centigrade around neurons and may lead to significant thermal effects on neuronal activity 20, 33, 34. Thus, the duration of LIFU-stim should be limited to minimize the thermal effect of continuous LIFU-stim pulses. A cumulative temperature variation equal to or greater than one degree centigrade in the right hippocampal CA1 region following LIFU stimulation was considered to indicate a thermal effect. We monitored the temperature variation in the right hippocampal CA1 region upon application of LIFU-stim **(Figure S6A)**. First, we observed no significant temperature variation (relative to baseline) in the CA1 region upon single-pulse LIFU stimulation **(Figure S6B)**. Then, continuous pulses of LIFU-stim at 15 s intervals were applied, and we observed an increase in the cumulative temperature variation in the CA1 region as the duration of the LIFU-stim increased **(Figure S6C-D)**. At a simulation time of 305 s, the maximum temperature variation in the right hippocampal CA1 region reached one degree centigrade **(Figure S6D)**; thus, to maximize the duration of LIFU stimulation while minimizing its thermal effects, continuous pulses of LIFU stimulation were applied at an interval of 15 s for 290 s **(Figure S6D)**.

**Effects of LIFU stimulation on mouse brain morphology and cognitive function.** According to the results of the subsequent morphologic investigation, 290 s of continuous pulse LIFU stimulation did not lead to obvious changes in the brain structure (e.g., right hippocampus and cortex) or cellular morphology (including neurons, astrocytes, and microglia) (**Figure S7**). Moreover, the results of the Morris water maze test indicated that LIFU stimulation did not cause cognitive impairment (**Figure S8**).

Based on the abovementioned data, continuous pulses of LIFU-stim were applied at an interval of 15 s for 290 s in subsequent studies.

### Ultrasound stimulation alone was unable to alleviate SE effectively

The latency of SE is considered a key factor affecting the generation and development of SE in clinical and animal conditions 35, 36. Thus, we believe that performing interventions (activating GABAergic neurons in the CA1 region) during the latency of SE may alleviate SE. We first investigated the effect of ultrasound stimulation alone on the latency of GSs prior to the induction of SE by KA (**Figure S9A**). We found that there was no significant difference in the latency to generalized seizures (GSs), latency to SE, or percentage of GSs between the sham group and the LIFU-stim group (**Figure S9B-D**), indicating that ultrasound stimulation alone did not ameliorate SE effectively.

### MG-SOG was unable to ameliorate SE effectively by activating SST-INs

An increase in the inhibitory effect of GABA-INs is beneficial for relieving SE 5. Thus, we assumed that targeted activation of GABA-INs in the CA1 region by the MG-SOG, in the latency of GSs prior to the occurrence of SE, may suppress or ameliorate SE. Somatostatin interneurons (SST-INs) and parvalbumin interneurons (PV-INs), which target the distal dendrites and perisomatic regions of ENs, respectively, to exert their synaptic inhibitory effects on ENs, are two major subtypes of GABA-INs in the hippocampal CA1 region 37, 38.

**Figure 2:**
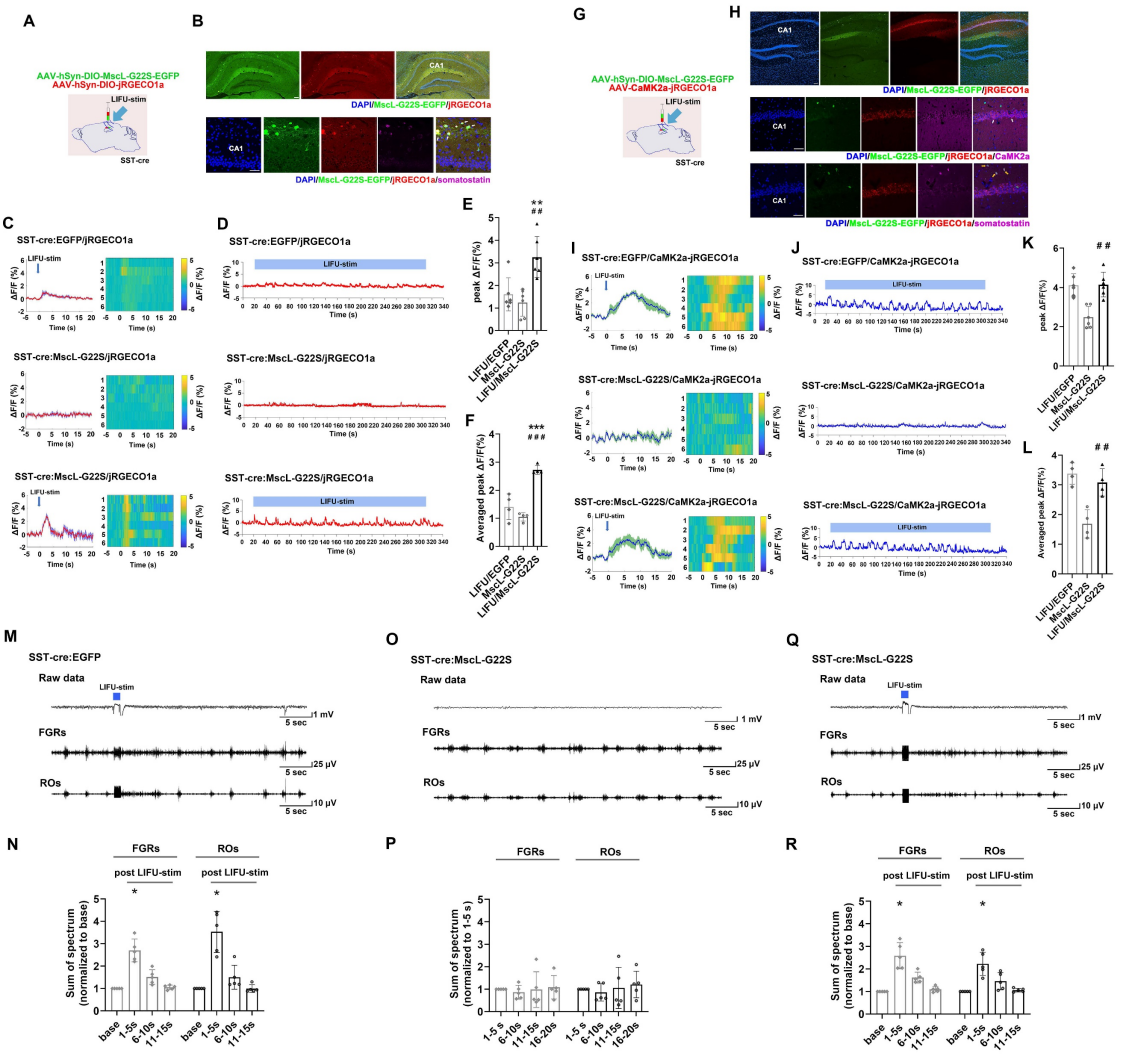
Neuronal calcium signals and LFPs in the hippocampal CA1 region upon MG-SOG-mediated activation of SST-INs. **(A)** Schematic diagram of stereotactic injection of AAV-hSyn-DIO-MscL-G22S-EGFP and AAV-hSyn-DIO-jRGECO1a into the hippocampal CA1 region of SST-cre mice for fiber photometry monitoring of the calcium signal in SST-INs activated by MG-SOG. **(B)** Representative fluorescence images of SST-INs coexpressing MscL-G22S-EGFP and jRGECO1a in the hippocampus (upper: *scale bar*, 100 μm) and immunofluorescence images of somatostatin-positive cells (labeled SST-INs) expressing MscL-G22S-EGFP and jRGECO1a in the CA1 region; the white arrows indicate the colocalization of MscL-G22S-EGFP, jRGECO1a, and somatostatin (lower: *scale bar*, 50 μm). **(C)** Calcium signals in SST-INs (plots of average values and heatmaps of ΔF/F, %) in the CA1 region upon application of a single pulse of LIFU-stim and** (D)** calcium signals in SST-INs (ΔF/F, %; representative trace) in the CA1 region upon application of continuous pulses of LIFU-stim. **(E)** The comparison of calcium signals (peak ΔF/F, %) in SST-INs upon application of a single pulse of LIFU-stim (6 tests for 3 mice per group), and **(F)** the comparison of calcium signals (average peak ΔF/F, %) in SST-INs upon application of continuous pulses of LIFU-stim (n = 4), among the LIFU/EGFP group, the MscL-G22S group (MscL-G22S alone), and the LIFU/MscL-G22S group (MscL-G22S expression with LIFU-stim); the data are presented as the mean ± SD, one-way ANOVA followed by Bonferroni *post hoc* test; **P < 0.01, ***P < 0.001, LIFU/MscL-G22S group compared to LIFU/EGFP group; ^##^*P* < 0.01, ^###^*P* < 0.001, LIFU/MscL-G22S group compared to MscL-G22S group. **(G)** Schematic diagram of stereotactic injection of AAV-hSyn-DIO-MscL-G22S-EGFP and AAV-CaMK2a-jRGECO1a into the hippocampal CA1 region of SST-cre mice for fiber photometry monitoring of calcium signals in ENs upon MG-SOG-mediated activation of SST-INs. **(H)** Representative fluorescence images of SST-INs expressing MscL-G22S-EGFP and ENs expressing jRGECO1a in the hippocampus (upper:* scale bar*, 100 μm). Immunofluorescence images of ENs coexpressing CaMK2a (ENs) and jRGECO1a and SST-INs expressing MscL-G22S-EGFP in the CA1 region; the white arrows indicate the colocalization of jRGECO1a and CaMK2a (middle: *scale bar*, 50 μm). Immunofluorescence images of SST-INs coexpressing somatostatin (SST-INs) and MscL-G22S-EGFP and ENs expressing jRGECO1a in the CA1 region; the yellow arrows indicate the colocalization of MscL-G22S-EGFP and somatostatin (lower: *scale bar*, 50 μm). **(I)** Calcium signals in ENs (plots of average values and heatmaps of ΔF/F, %) in the CA1 region upon application of a single pulse of LIFU-stim, and **(J)** calcium signal in ENs (ΔF/F, %; representative trace) in the CA1 region upon application of continuous pulses of LIFU-stim. **(K)** The comparison of calcium signals (peak ΔF/F, %) in ENs upon application of a single pulse of LIFU-stim (6 tests for 3 mice per group), and **(L)** the comparison of calcium signals (average peak ΔF/F, %) in ENs upon application of continuous pulses of LIFU-stim (n = 4), among the LIFU/EGFP group, the MscL-G22S group, and the LIFU/MscL-G22S group; the data are presented as the mean ± SD, one-way ANOVA followed by Bonferroni *post hoc* test; ^##^*P* < 0.01, LIFU/MscL-G22S group compared to MscL-G22S group. **(M)** Representative traces, including raw data and FGRs and ROs, of LFP recordings of the hippocampal CA1 region in the LIFU/EGFP group. **(N)** The sums of spectra of FGRs and ROs (values measured 1-5 sec, 6-10 sec, and 11-15 sec after LIFU-stim were normalized to baseline values) in the LIFU/EGFP group (n = 5). **(O)** Representative traces, including raw data and FGRs and ROs, of LFP recordings of the hippocampal CA1 region in the MscL-G22S group (without LIFU stimulation). **(P)** The sums of spectra of FGRs and ROs (values measured 6-10 sec, 11-15 sec, and 16-20 sec were normalized to those measured 1-5 sec (base)) in the MscL-G22S group (n = 5). **(Q)** Representative traces, including raw data and FGRs and ROs, of LFP recordings of the hippocampal CA1 region in the LIFU/MscL-G22S group. **(R)** The sums of spectra of FGRs and ROs (values measured 1-5 sec, 6-10 sec, and 11-15 sec after LIFU-stim were normalized to baseline values) in the LIFU/MscL-G22S group (n = 5). One-way RM-ANOVA followed by Bonferroni *post hoc* test in N, P, and R; *P < 0.05, 1-5 sec after LIFU-stim compared to baseline.

First, we aimed to determine whether MG-SOG could activate SST-INs effectively. For this purpose, AAV-hSyn-DIO-MscL-G22S-EGFP and AAV-hSyn-DIO-jRGECO1a (a red fluorescent calcium indicator) 39 were simultaneously injected into the right CA1 region of SST-cre mice **(Figure 2A)** to induce the expression of both MscL-G22S and jRGECO1a in SST-INs. After 4 weeks, we confirmed the colocalization of MscL-G22S and jRGECO1a in SST-INs in the right hippocampus via immunohistochemistry **(Figure 2B)**. Fiber photometry was subsequently used to measure whether MG-SOG could activate SST-INs effectively, which demonstrated that MG-SOG (the synergy of LIFU-stim and SST-expressing MscL-G22S) increased the Ca^2+^ response of SST-INs to both a single pulse of LIFU-stim (**Figure 2C, E**) and continuous pulses of LIFU-stim (**Figure 2D, F**), indicating that MG-SOG could effectively promote the activation of SST-INs.

Second, we investigated the effect of MG-SOG-mediated activation of SST-INs on the Ca^2+^ response of ENs in the CA1 region. For this purpose, AAV-hSyn-DIO-MscL-G22S-EGFP and AAV-CaMK2α-jRGECO1a were simultaneously injected into the CA1 region of SST-Cre mice **(Figure 2G)** to induce the expression of MscL-G22S in SST-INs and jRGECO1a in ENs, respectively **(Figure 2H)**. Subsequent fiber photometry analyses demonstrated that activation of SST-INs by MG-SOG did not significantly reduce calcium signaling in ENs **(Figure 2I-L)**, indicating that MG-SOG-mediated activation of SST-INs had little impact on controlling EN excitability. LFP recordings from the CA1 region revealed that in both the LIFU/EGFP group (LIFU-stim alone) **(Figure 2M-N)** and the LIFU/MscL-G22S group (MG-SOG) **(Figure 2Q-R)**, the sums of the spectra of FGRs and ROs in the CA1 region were significantly increased upon application of a single pulse of LIFU-stim, indicating that MG-SOG-mediated activation of SST-INs did not control the excitability of the LNN in the CA1 region effectively. Without LIFU stimulation, SST-INs expressing MscL-G22S alone had no significant effect on the FGRs or ROs in the CA1 region **(Figure 2O-P)**.

**Figure 3:**
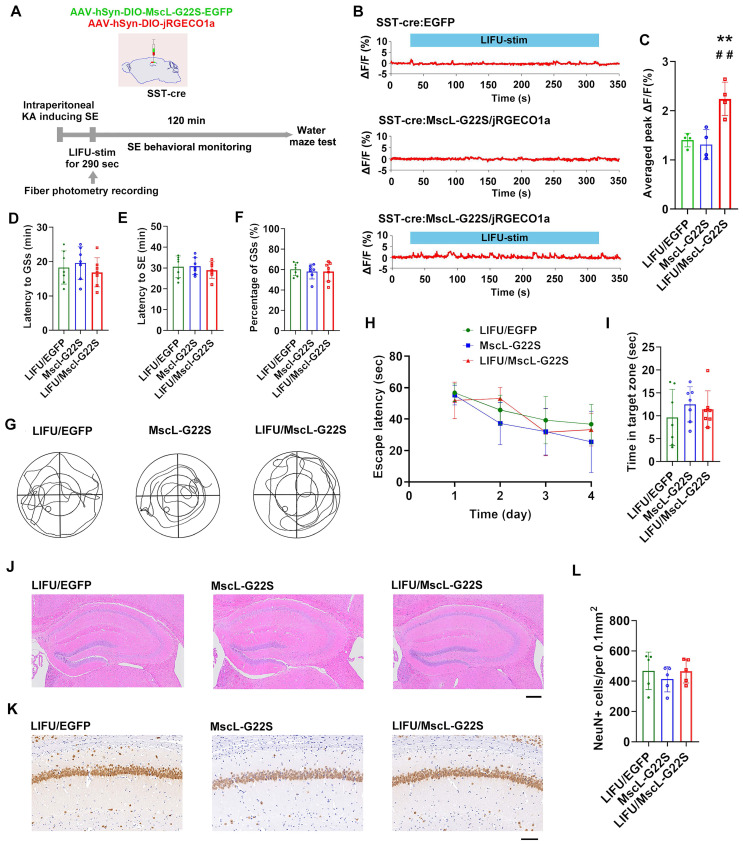
The effects of MG-SOG-mediated activation of SST-INs on KA-induced SE and SE-related cognitive impairment. **(A)** Schematic of the experimental design. After stereotactic injection of AAV-hSyn-DIO-MscL-G22S-EGFP and AAV-hSyn-DIO-jRGECO1a into the hippocampal CA1 region of SST-cre mice, followed by induction of SE by KA, and then continuous pulses of LIFU-stim were applied in the latency of GSs, followed by SE behavioral monitoring; after 1 week of KA-induced SE, Morris water maze tests were performed to assess cognitive function. **(B)** Calcium signals in SST-INs (ΔF/F, %; representative trace) in the CA1 region upon application of continuous pulses of LIFU-stim in the latency of GSs from LIFU/EGFP group, MscL-G22S group, and LIFU/MscL-G22S group. **(C)** The comparison of calcium signals (average peak ΔF/F, %) in SST-INs upon application of continuous pulses of LIFU-stim (n = 4), among the LIFU/EGFP group, the MscL-G22S group, and the LIFU/MscL-G22S group. **(D)** Comparisons of the latency to GSs (min), **(E)** latency to SE (min), and **(F)** percentage of GSs (%) among LIFU/EGFP group, MscL-G22S group, and LIFU/MscL-G22S group (n = 7). The Morris water maze test was used for evaluating cognitive function: **(G)** the representative trajectoris of mice from LIFU/EGFP group, MscL-G22S group, and LIFU/MscL-G22S group, **(H)** the escape latency (sec) over four training days (n = 7), and **(I)** time spent in the target zone (n = 7). **(J)** Hematoxylin-eosin staining of mouse brains (*scale bar*, 200 μm) and **(K)** immunohistochemical staining of NeuN (neuronal marker) (*scale bar*, 100 μm) from LIFU/EGFP group, MscL-G22S group, and LIFU/MscL-G22S group; **(L)** comparison of the number of NeuN+ cells among the LIFU/EGFP group, the MscL-G22S group, and the LIFU/MscL-G22S group (n = 5). One-way ANOVA followed by Bonferroni *post hoc* test in C, D, E, F, I, and L, **P < 0.01, LIFU/MscL-G22S group compared to LIFU/EGFP group; ^##^*P* < 0.01, LIFU/MscL-G22S group compared to MscL-G22S group; two-way RM-ANOVA followed by Bonferroni *post hoc* test in H; the data are presented as the mean ± SD.

Third, we investigated the potential roles of MG-SOG in activating SST-INs in SE model mice **(Figure 3A)**. The MG-SOG activated SST-INs effectively during the latency of the GSs prior to the occurrence of SE (**Figure 3B-C**). SE behavioral monitoring revealed that there was no significant difference in the latency to GSs, latency to SE, or percentage of GSs among the LIFU/EGFP group, the MscL-G22S group (MscL-G22S alone), or the LIFU/MscL-G22S group (**Figure 3D-F**), indicating that the activation of SST-INs by MG-SOG did not effectively inhibit SE. SE is closely associated with cognitive impairment 40-42. The Morris water maze test was subsequently used to evaluate cognitive function after SE (1 week after KA-induced SE). There was no significant difference in the escape latency over four training days or time spent in the target zone among the three groups (**Figure 3G-I**), indicating that MG-SOG-mediated activation of SST-INs during the latency of SE had no significant effect on cognitive function after KA-induced SE. Hematoxylin‒eosin (HE) staining of the hippocampus indicated that MG-SOG-mediated activation of SST-INs did not lead to obvious changes in the hippocampal structure (**Figure 3J**); immunohistochemical staining of NeuN in the hippocampal CA1 region indicated that MG-SOG-mediated activation of SST-INs did not change the number of NeuN+ cells in the CA1 region (**Figure 3K-L**).

**Figure 4:**
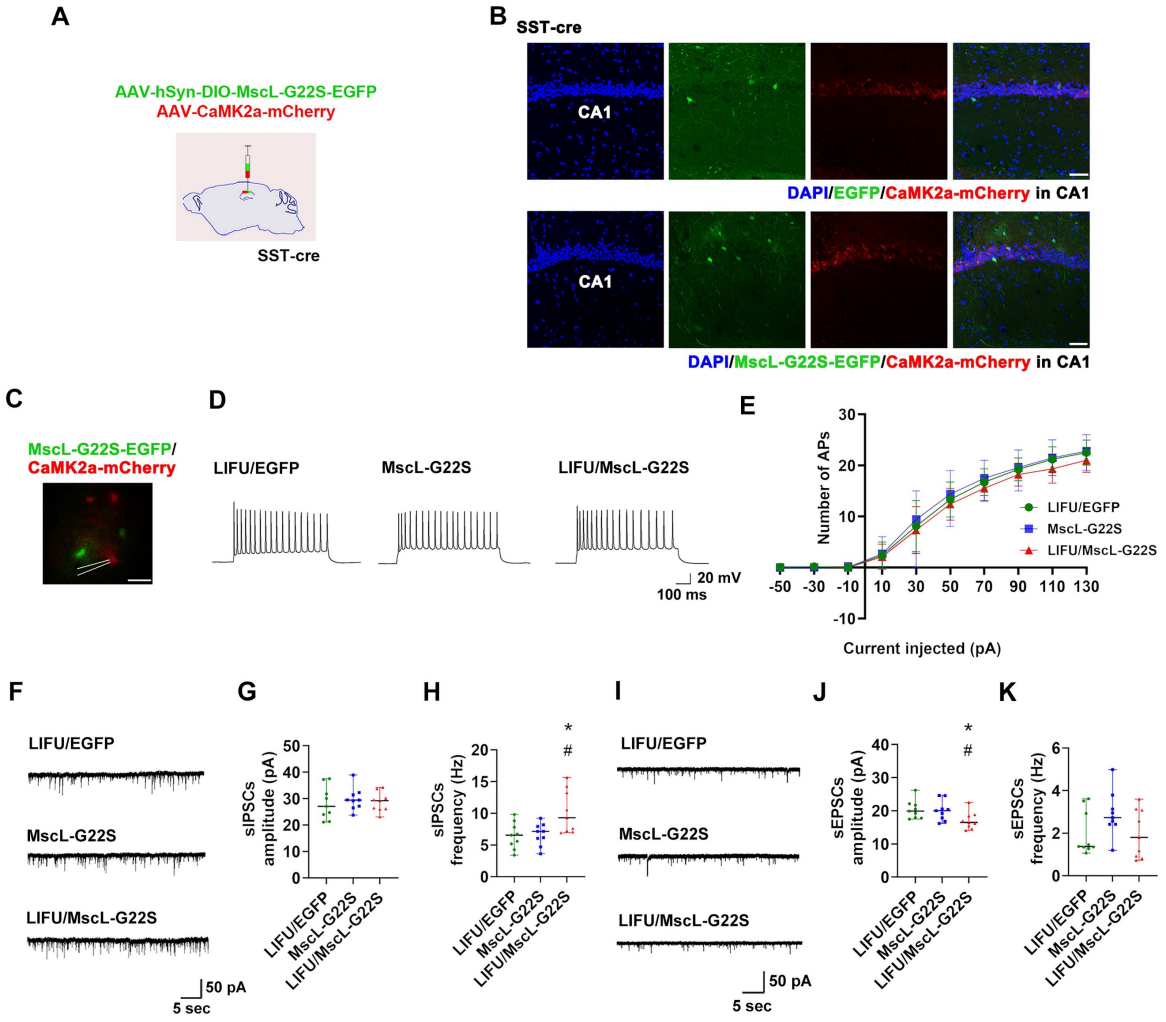
The effects of MG-SOG-mediated activation of SST-INs on the SE-related electrophysiological properties of ENs in hippocampal CA1 region of KA-induced SE model mice. **(A)** Schematic of the experimental design: after stereotactic injection of AAV-hSyn-DIO-MscL-G22S-EGFP and AAV-CaMK2a-mCherry into the hippocampal CA1 region of SST-cre mice, followed by induction of SE by KA; after 2 hours of KA-induced SE, patch clamp recordings were performed to test electrophysiological properties of ENs. **(B)** Representative fluorescence images of SST-INs expressing EGFP or MscL-G22S-EGFP, and CaMK2a+ ENs expressing mCherry in the hippocampus (*scale bar*, 50 μm). **(C)** Representative fluorescence image of ENs expressing CaMK2a-mCherry and SST-INs expressing MscL-G22S-EGFP during patch clamp recording (*scale bar*, 20 μm) and **(D)** representative traces of EN APs from LIFU/EGFP group, MscL-G22S group, and LIFU/MscL-G22S group; **(E)** the comparison of the EN AP number versus injected current curve among the three groups (n = 10 cells per group from 3 mice). **(F)** Representative traces of sIPSCs in ENs from LIFU/EGFP group, MscL-G22S group, and LIFU/MscL-G22S group, and comparisons of **(G)** the amplitude and** (H)** frequency of sIPSCs of ENs among the three groups (n = 9 cells per group from 3 mice). **(I)** Representative traces of sEPSCs in ENs from LIFU/EGFP group, MscL-G22S group, and LIFU/MscL-G22S group, and comparisons of **(J)** the amplitude and** (K)** frequency of sEPSCs of ENs among the three groups (n = 9 cells per group from 3 mice). Two-way RM-ANOVA followed by Bonferroni *post hoc* test in E, the data are the mean ± SD; one-way nonparametric kruskal-wallis ANOVA test in G, H, J, K, the data are median and range; *P < 0.05, LIFU/MscL-G22S group compared to LIFU/EGFP group; ^#^*P* < 0.05, LIFU/MscL-G22S group compared to MscL-G22S group.

Next, patch clamp recording was used to investigate the effects of MG-SOG-mediated activation of SST-INs on the SE-related electrophysiological properties of ENs in the hippocampal CA1 region in these SE model mice. To simultaneously induce the expression of MscL-G22S in SST-INs and label ENs, AAV-hSyn-DIO-MscL-G22S-EGFP and AAV-CaMK2α-mCherry were injected into the CA1 region of SST-Cre mice (**Figure 4A-B**), followed by the induction of SE by KA; after KA-induced SE, patch clamp recordings of ENs in the hippocampal CA1 region were performed, which demonstrated that there was no significant difference in the number of APs produced by ENs among the LIFU/EGFP group, the MscL-G22S group, and the LIFU/MscL-G22S group (**Figure 4C-E;** details of the intrinsic physiological properties of APs produced by ENs are listed in **Table S2**). Moreover, the frequency of sIPSCs in ENs was increased in SST-INs activated by MG-SOG **(Figure 4F, H)**, whereas the amplitude of sEPSCs was reduced **(Figure 4I, J)**; however, MG-SOG did not alter the amplitude of sIPSCs **(Figure 4G)** and the frequency of sEPSCs **(Figure 4K)**. These data indicated that MG-SOG-mediated activation of SST-INs did not obviously alleviate SE-related hyperexcitability or the EI-IM of ENs in the hippocampal CA1 region in SE.

### MG-SOG effectively inhibited SE initiation by activating PV-INs

**Figure 5:**
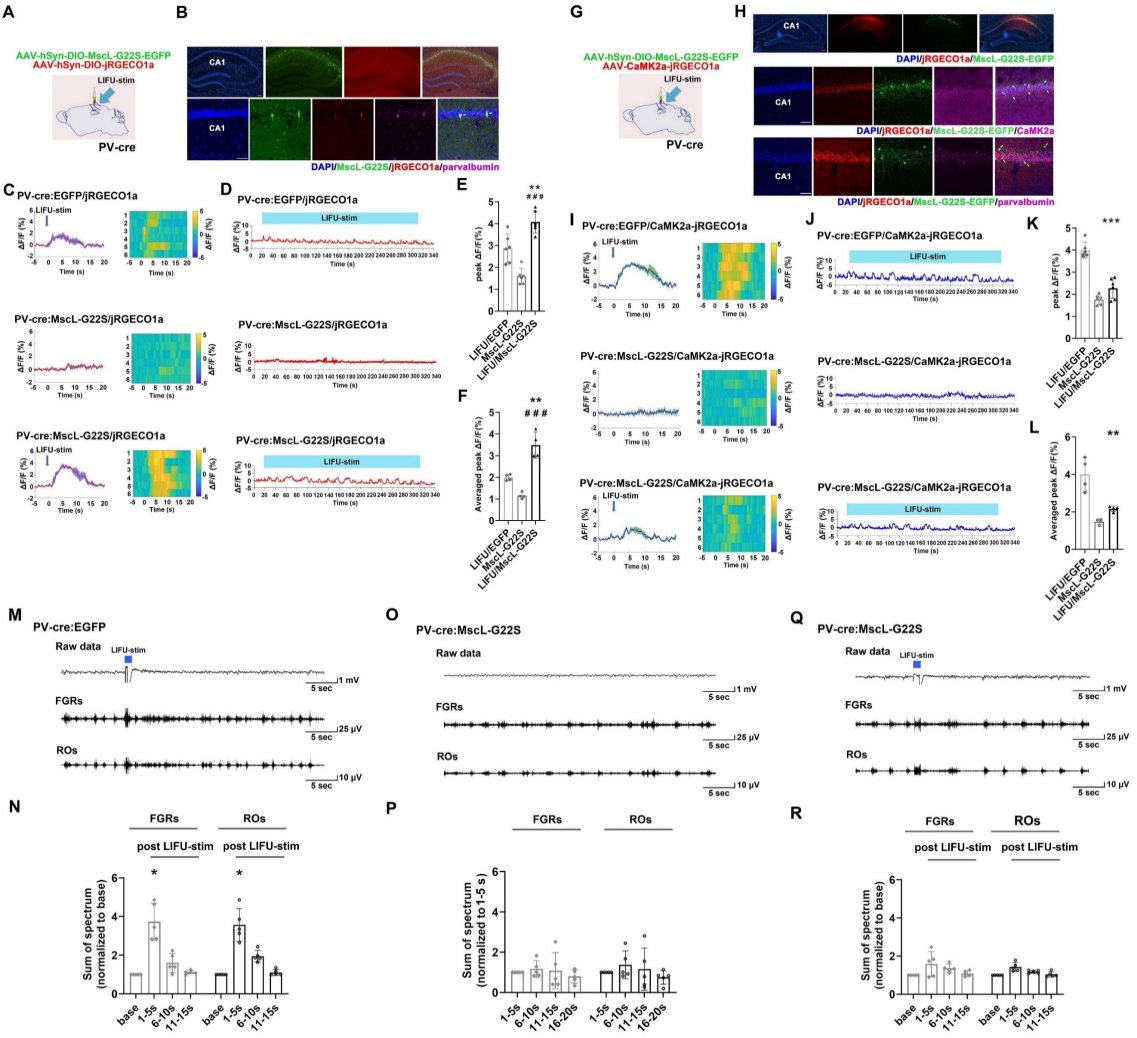
Neuronal calcium signals and LFPs in the hippocampal CA1 region upon MG-SOG-mediated activation of PV-INs. **(A)** Schematic diagram of stereotactic injection of AAV-hSyn-DIO-MscL-G22S-EGFP and AAV-hSyn-DIO-jRGECO1a into the hippocampal CA1 region of PV-cre mice for fiber photometry monitoring of the calcium signal in PV-INs activated by MG-SOG. **(B)** Representative fluorescence images of PV-INs coexpressing MscL-G22S-EGFP and jRGECO1a in the hippocampus (upper: *scale bar*, 100 μm) and immunofluorescence images of parvalbumin-positive cells (labeled PV-INs) expressing MscL-G22S-EGFP and jRGECO1a in the CA1 region; the white arrows indicate the colocalization of MscL-G22S-EGFP, jRGECO1a, and parvalbumin (lower: *scale bar*, 50 μm). **(C)** Calcium signals in PV-INs (plots of average values and heatmaps of ΔF/F, %) in the CA1 region upon application of a single pulse of LIFU-stim and **(D)** the calcium signals in PV-INs (ΔF/F, %; representative trace) in the CA1 region upon application of continuous pulses of LIFU-stim. **(E)** The comparison of calcium signals (peak ΔF/F, %) in PV-INs upon application of a single pulse of LIFU-stim (6 tests for 3 mice per group) and **(F)** the comparison of calcium signals (average peak ΔF/F, %) in PV-INs upon application of continuous pulses of LIFU-stim (n = 4), among the LIFU/EGFP group, the MscL-G22S group (MscL-G22S alone), and the LIFU/MscL-G22S group (MscL-G22S expression with LIFU-stim); the data are presented as the mean ± SD, one-way ANOVA followed by Bonferroni *post hoc* test; **P < 0.01, LIFU/MscL-G22S group compared to LIFU/EGFP group; ^###^*P* < 0.001, LIFU/MscL-G22S group compared to MscL-G22S group. **(G)** Schematic diagram of stereotactic injection of AAV-hSyn-DIO-MscL-G22S-EGFP and AAV-CaMK2a-jRGECO1a into the hippocampal CA1 region of PV-cre mice for fiber photometry monitoring of calcium signals in ENs upon MG-SOG-mediated activation of PV-INs. **(H)** Representative fluorescence images of PV-INs expressing MscL-G22S-EGFP and ENs expressing jRGECO1a in the hippocampus (upper: *scale bar*, 100 μm). Immunofluorescence images of ENs coexpressing CaMK2a and jRGECO1a and PV-INs expressing MscL-G22S-EGFP in the CA1 region; the white arrows indicate the colocalization of jRGECO1a and CaMK2a (middle: *scale bar*, 50 μm). Immunofluorescence images of PV-INs coexpressing parvalbumin and MscL-G22S-EGFP and ENs expressing jRGECO1a in the CA1 region; the yellow arrows indicate the colocalization of MscL-G22S-EGFP and parvalbumin (lower: *scale bar*, 50 μm). **(I)** Calcium signals in ENs (plots of average values and heatmaps of ΔF/F, %) in the CA1 region upon application of a single pulse of LIFU-stim and** (J)** calcium signal in ENs (ΔF/F, %; representative trace) in the CA1 region upon application of continuous pulses of LIFU-stim. **(K)** The comparison of calcium signals (peak ΔF/F, %) in ENs upon application of a single pulse of LIFU-stim (6 tests for 3 mice per group) and **(L)** the comparison of calcium signals (average peak ΔF/F, %) in ENs upon application of continuous pulses of LIFU-stim (n = 4), among the LIFU/EGFP group, the MscL-G22S group, and the LIFU/MscL-G22S group. The data are presented as the mean ± SD, one-way ANOVA followed by Bonferroni *post hoc* test; **P < 0.01, ***P < 0.001, LIFU/MscL-G22S group compared to LIFU/EGFP group. **(M)** Representative traces, including raw data and FGRs and ROs, of LFP recordings of the hippocampal CA1 region in the LIFU/EGFP group. **(N)** The sums of spectra of FGRs and ROs (values measured 1-5 sec, 6-10 sec, and 11-15 sec after LIFU-stim were normalized to baseline values) in the LIFU/EGFP group (n = 5). **(O)** Representative traces, including raw data and FGRs and ROs, of LFP recordings of the hippocampal CA1 region in the MscL-G22S group. **(P)** The sums of spectra of FGRs and ROs (values measured 6-10 sec, 11-15 sec, and 16-20 sec were normalized to those measured 1-5 sec (base)) in the MscL-G22S group (n = 5). **(Q)** Representative traces, including raw data and FGRs and ROs, of LFP recordings of the hippocampal CA1 region in the LIFU/MscL-G22S group. **(R)** The sums of spectra of FGRs and ROs (values measured 1-5 sec, 6-10 sec, and 11-15 sec after LIFU-stim were normalized to baseline values) in the LIFU/MscL-G22S group (n = 5). One-way RM-ANOVA followed by Bonferroni *post hoc* test in N, P, and R; *P < 0.05, 1-5 sec after LIFU-stim compared to baseline.

To determine the effects of MG-SOG on the activity of PV-INs, AAV-hSyn-DIO-MscL-G22S-EGFP and AAV-hSyn-DIO-jRGECO1a were simultaneously injected into the right CA1 region of PV-Cre mice to induce the expression of both MscL-G22S and jRGECO1a in PV-INs **(Figure 5A)**. After 4 weeks, the colocalization of MscL-G22S and jRGECO1a in PV-INs in the right hippocampus was detected **(Figure 5B)**; subsequent analyses of fiber photometry data demonstrated that MG-SOG (the synergy of LIFU-stim and PV-INs expressing MscL-G22S) increased the Ca^2+^ response of PV-INs upon application of both a single pulse of LIFU-stim (**Figure 5C, E**) and continuous pulses of LIFU-stim (**Figure 5D, F**), indicating that MG-SOG could activate PV-INs effectively.

We further evaluated the effect of the MG-SOG-mediated activation of PV-INs on Ca^2+^ signaling in ENs. AAV-hSyn-DIO-MscL-G22S-EGFP and AAV-CaMK2α-jRGECO1 were simultaneously injected into the CA1 region of PV-cre mice **(Figure 5G)** to induce the expression of MscL-G22S in PV-INs and jRGECO1a in ENs **(Figure 5H)**. Fiber photometry demonstrated that MG-SOG significantly decreased calcium signaling in ENs, indicating that the MG-SOG-mediated activation of PV-INs decreased the excitability of ENs; without LIFU stimulation, PV-INs expressing MscL-G22S alone had no significant effect on the excitability of ENs in the CA1 region **(Figure 5I-L)**. LFP recordings of the CA1 region revealed that the sums of spectra of FGRs and ROs in the CA1 region were increased in the EGFP group upon application of a single pulse of LIFU-stim (LIFU-EGFP group) **(Figure 5M-N)**; however, in the LIFU/MscL-G22S group, the sums of spectra of FGRs and ROs in the CA1 region did not increase upon application of a single pulse of LIFU-stim **(Figure 5Q-R)**, which indicated that MG-SOG-mediated activation of PV-INs could control the excitability of the LNN in the CA1 region; without LIFU-stim, PV-INs expressing MscL-G22S alone had no significant effect on the FGRs or ROs in the CA1 region **(Figure 5O-P)**.

**Figure 6:**
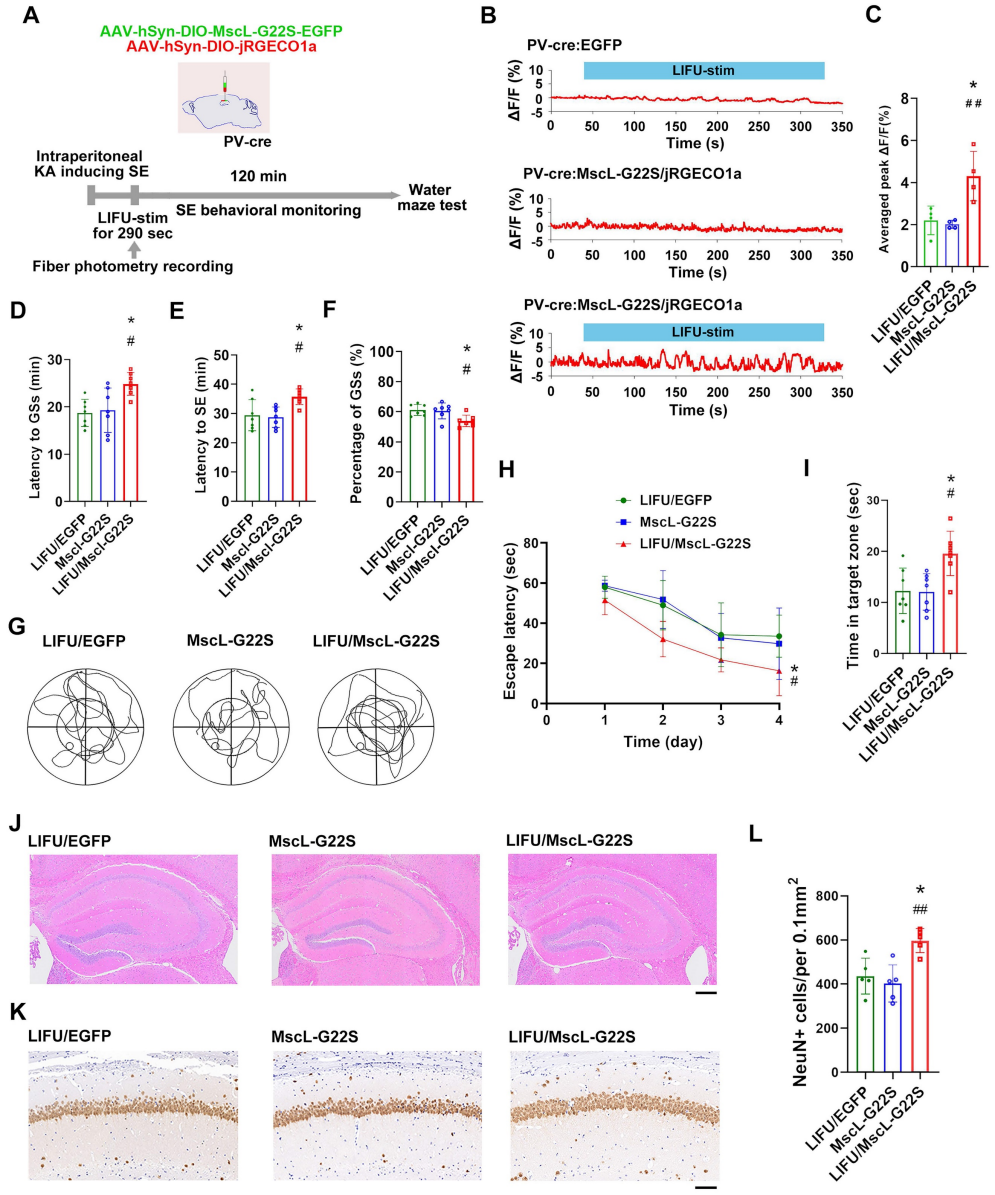
The effects of MG-SOG-mediated activation of PV-INs on KA-induced SE and SE-related cognitive impairment. **(A)** Schematic of the experimental design. After stereotactic injection of AAV-hSyn-DIO-MscL-G22S-EGFP and AAV-hSyn-DIO-jRGECO1a into the hippocampal CA1 region of PV-cre mice, followed by induction of SE by KA, and then continuous pulses of LIFU-stim were applied in the latency of GSs, followed by SE behavioral monitoring; after 1 week of KA-induced SE, Morris water maze tests were performed to assess cognitive function. **(B)** Calcium signals in PV-INs (ΔF/F, %; representative trace) in the CA1 region upon application of continuous pulses of LIFU-stim in the latency of GSs from LIFU/EGFP group, MscL-G22S group, and LIFU/MscL-G22S group. **(C)** The comparison of calcium signals (average peak ΔF/F, %) in PV-INs upon application of continuous pulses of LIFU-stim (n = 4), among the LIFU/EGFP group, the MscL-G22S group, and the LIFU/MscL-G22S group. **(D)** Comparisons of the latency to GSs (min), **(E)** latency to SE (min), and **(F)** percentage of GSs (%) among LIFU/EGFP group, MscL-G22S group, and LIFU/MscL-G22S group (n = 7). The Morris water maze test was used for evaluating cognitive function: **(G)** the representative trajectoris of mice from LIFU/EGFP group, MscL-G22S group, and LIFU/MscL-G22S group, **(H)** the escape latency (sec) over four training days (n = 7), and **(I)** time spent in the target zone (n = 7). **(J)** Hematoxylin-eosin staining of mouse brains (*scale bar*, 200 μm) and **(K)** immunohistochemical staining of NeuN (neuronal marker) (*scale bar*, 100 μm) from LIFU/EGFP group, MscL-G22S group, and LIFU/MscL-G22S group; **(L)** comparison of the number of NeuN+ cells among LIFU/EGFP group, MscL-G22S group, and LIFU/MscL-G22S group (n = 5). One-way ANOVA followed by Bonferroni *post hoc* test in C, D, E, F, I, and L; two-way RM-ANOVA followed by Bonferroni *post hoc* test in H; the data are presented as the mean ± SD; *P < 0.05, LIFU/MscL-G22S group compared to LIFU/EGFP group;^ #^*P* < 0.05, ^##^*P* < 0.01, LIFU/MscL-G22S group compared to MscL-G22S group.

Next, we investigated the potential roles of MG-SOG in activating PV-INs in SE model mice **(Figure 6A)**. The MG-SOG effectively activated the PV-INs during the latency of the GSs prior to the occurrence of SE (**Figure 6B-C**).

SE behavioral monitoring revealed that in the LIFU/MscL-G22S group, the latency to GSs and latency to SE were prolonged and the percentage of GSs was reduced (**Figure 6D-F**), indicating that the activation of PV-INs by MG-SOG alleviated SE. The Morris water maze test was subsequently used to evaluate cognitive function after SE, which revealed that the escape latency over four training days was reduced and that the time spent in the target zone was increased in the LIFU/MscL-G22S group (**Figure 6G-I**), indicating that MG-SOG-mediated activation of PV-INs during the latency of SE improved cognitive function after KA-induced SE. HE staining of the hippocampus revealed that this effect did not lead to obvious changes in the hippocampal structure (**Figure 6J**); immunohistochemical staining of NeuN in the hippocampal CA1 region indicated that MG-SOG-mediated activation of PV-INs increased the number of NeuN+ cells in the CA1 region (**Figure 6K-L**), indicating that this intervention might play a neuroprotective role in SE.

**Figure 7:**
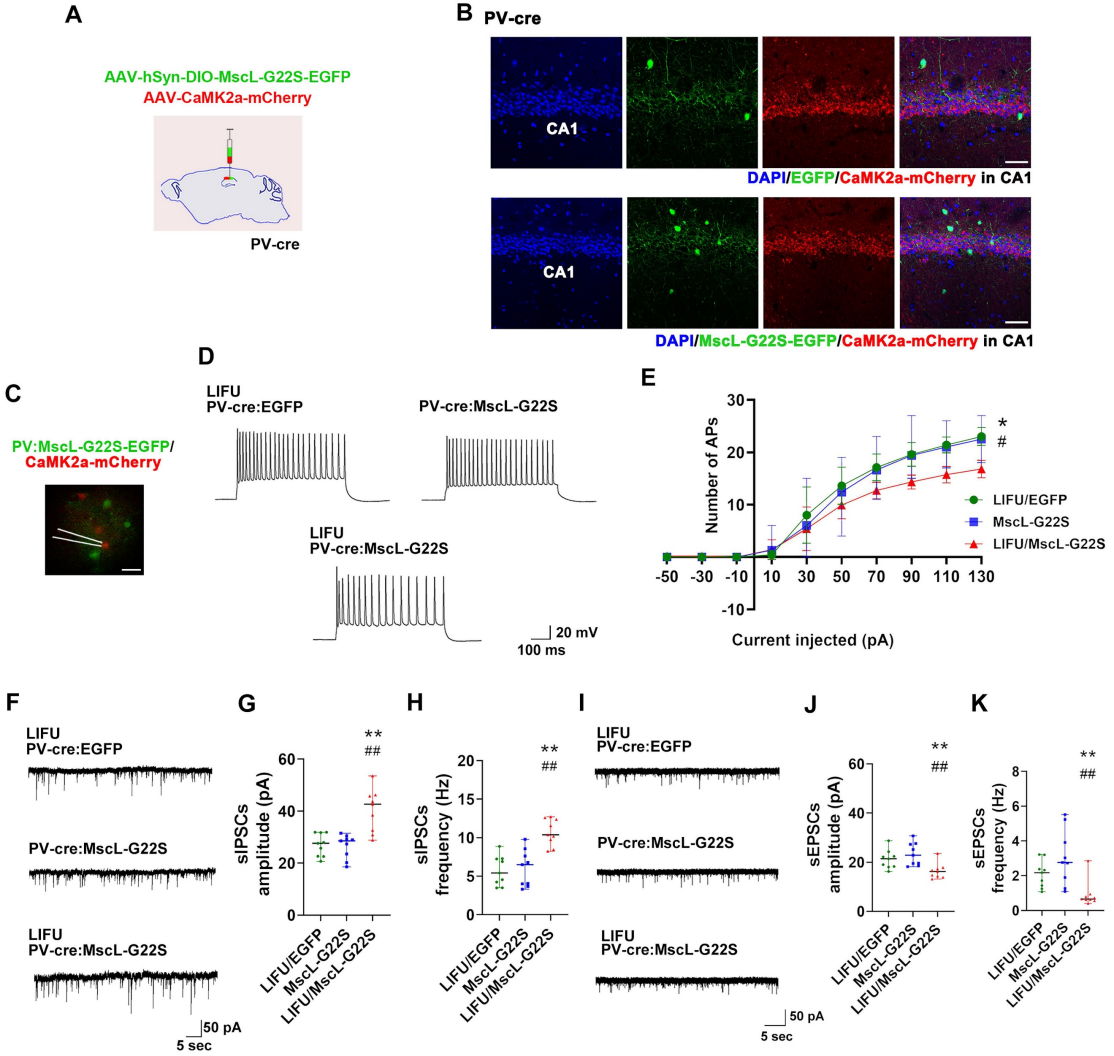
The effects of MG-SOG-mediated activation of PV-INs on the SE-related electrophysiological properties of ENs in hippocampal CA1 region of KA-induced SE model mice. **(A)** Schematic of the experimental design: after stereotactic injection of AAV-hSyn-DIO-MscL-G22S-EGFP and AAV-CaMK2a-mCherry into the hippocampal CA1 region of PV-cre mice, followed by induction of SE by KA; after 2 hours of KA-induced SE, patch clamp recordings were performed to test electrophysiological properties of ENs. **(B)** Representative fluorescence images of PV-INs expressing EGFP or MscL-G22S-EGFP, and CaMK2a+ ENs expressing mCherry in the hippocampus (*scale bar*, 50 μm). **(C)** Representative fluorescence image of ENs expressing CaMK2a-mCherry and PV-INs expressing MscL-G22S-EGFP during patch clamp recording (*scale bar,* 20 μm) and **(D)** representative traces of EN APs from LIFU/EGFP group, MscL-G22S group, and LIFU/MscL-G22S group; **(E)** the comparison of the EN AP number versus injected current curve among the three groups (n = 10 cells per group from 3 mice). **(F)** Representative traces of sIPSCs in ENs from LIFU/EGFP group, MscL-G22S group, and LIFU/MscL-G22S group, and comparisons of **(G)** the amplitude and** (H)** frequency of sIPSCs of ENs among the three groups (n = 9 cells per group from 3 mice). **(I)** Representative traces of sEPSCs in ENs from LIFU/EGFP group, MscL-G22S group, and LIFU/MscL-G22S group, and comparisons of **(J)** the amplitude and** (K)** frequency of sEPSCs of ENs among the three groups (n = 9 cells per group from 3 mice). Two-way RM-ANOVA followed by Bonferroni *post hoc* test in E, the data are the mean ± SD; one-way nonparametric kruskal-wallis ANOVA test in G, H, J, K, the data are median and range; *P < 0.05, **P < 0.01, LIFU/MscL-G22S group compared to LIFU/EGFP group; ^#^*P* < 0.05,^ ##^*P* < 0.01, LIFU/MscL-G22S group compared to MscL-G22S group.

Next, patch clamp recording was performed to determine the effects of MG-SOG-mediated PV-IN activation on the SE-related electrophysiological properties of ENs in the hippocampal CA1 region. First, to simultaneously induce the expression of MscL-G22S in PV-INs and label ENs, AAV-hSyn-DIO-MscL-G22S-EGFP and AAV-CaMK2α-mCherry were injected into the CA1 region of PV-Cre mice (**Figure 7A-B**), followed by the induction of SE by KA; then, patch clamp recordings of ENs in the hippocampal CA1 region were performed. In the LIFU/MscL-G22S group, the number of APs produced by ENs was significantly reduced (**Figure 7C-E;** details of the intrinsic physiological properties of APs produced by ENs are listed in **Table S3**). Moreover, the frequency and amplitude of sIPSCs in ENs were obviously increased in PV-INs activated by MG-SOG **(Figure 7F-H)**, whereas the frequency and amplitude of sEPSCs were significantly reduced **(Figure 7I-K)**. These data indicated that MG-SOG-mediated PV-IN activation alleviated SE-related hyperexcitability and the EI-IM of ENs in the hippocampal CA1 region in SE.

## Discussion

In the present study, we demonstrated that MG-SOG, an ultrasound neurostimulation approach utilizing the mechanosensitive ion channel MscL-G22S, could ameliorate the behavioral phenotypes of SE model mice by activating PV-INs in the hippocampal CA1 region during the latency of SE. This strategy may represent a sonogenetic neurostimulation approach for relieving SE and further help us understand the underlying mechanism of SE initiation and progression.

Accumulating evidence suggests that neuromodulatory treatments, such as deep brain stimulation and responsive neurostimulation, can regulate seizure activity in humans and animals 43-45. Prior to utilizing neuromodulatory strategies, it is important to find the optimal target brain region or nerve 43-45. The abnormal function of the hippocampal CA1 region was found to be associated with SE 26-28. Therefore, we focused on the activation of hippocampal neurons in the CA1 region in KA-induced SE model mice. Thus, we speculated that the hippocampal CA1 region might play a role in the initiation and progression of SE and selected it as the target brain region for subsequent LIFU-stim experiments. The hyperexcitability of ENs or insufficient inhibitory activity of GABA-INs may cause EI-IM, resulting in LNN hyperexcitability in specific brain regions and further causing or promoting seizure activity 46. Interestingly, we found that both ENs and GABA-INs in the CA1 region were activated in the SE group. Similarly, subsequent patch clamp recordings revealed EN hyperexcitability in the CA1 region in the SE group, with increased EST to ENs and IST from GABA-INs to ENs. Moreover, we also noted that the increase in EST was more obvious than the increase in IST, which may indicate that the EI-IM in the CA1 region was due to insufficient inhibitory effects of GABAergic-INs on ENs. Thus, we consider that activating GABA-INs during the latency of SE is an efficient way to increase GABAergic inhibitory activity and thus decrease the excitability of ENs, further inhibiting the initiation and progression of SE.

The appropriate LIFU-stim parameters should be determined prior to investigating the potential effect of the MG-SOG. Ultrasonic stimulation can produce multiple physical effects, including mechanical forces, thermal effects, and cavitation effects 47. Ultrasound-induced neuromodulation mainly results from mechanical forces from low acoustic pressure, which may also produce thermal or cavitation effects 19, 47. Thus, we used LIFU-stim to produce low acoustic pressure in this study. In this study, we used low-intensity ultrasonic stimulation, which produced a low acoustic pressure. Previous studies also indicated that this intensity of ultrasonic stimulation did not produce an obvious cavitation effect 24, 48. Moreover, the obvious cavitation effect caused by ultrasonic stimulation usually results in obvious structural or cellular alternations in the local region; thus, we performed morphological experiments to assess whether the LIFU-stim strategy used in this study could cause obvious structural or cellular alterations in the CA1 region, which showed that this LIFU-stim strategy did not cause structural or cellular alterations directly. We also performed HE staining to assess whether there was a histological structural change upon MG-SOG-mediated SST-IN or PV-IN activation, which indicated that MG-SOG-mediated SST-IN or PV-IN activation did not cause significant histological structural alterations in the CA1 region. Moreover, the thermal effect of ultrasonic stimulation is determined by the stimulation intensity or by the stimulation duration when the stimulation intensity is low. Therefore, we selected an adequate stimulation duration to produce sufficient mechanical force while also limiting the stimulation duration to avoid obvious thermal effects in this study. Additionally, we investigated the ability of LIFU stimulation alone to stimulate the CA1 region. We found that LIFU stimulation alone effectively promoted neuronal activation in the CA1 region and that this effect was not specific to ENs or GABA-INs. We also noted that LIFU-stim preferentially affected ENs, possibly increasing the excitability of the LNN in the CA1 region. We assumed that LIFU stimulation alone might not relieve EI-IM in the CA1 region in SE model mice, and subsequent investigations revealed that LIFU stimulation alone did not restrain the initiation or progression of SE. On the basis of these data, LIFU stimulation alone was unable to effectively alleviate SE because of its lack of specificity for neurons (ENs or GABA-INs).

Sonogenetics can increase the brain region specificity of LIFU stimulation and allow LIFU stimulation to achieve cell type specificity by promoting the expression of mechanosensitive ion channels within a brain region 22, 24. In this study, we investigated the effects of sonogenetics-mediated activation of SST-INs and PV-INs in the CA1 region. Upon treatment with SST-INs expressing MscL-G22S, MG-SOG could activate SST-INs effectively; however, MG-SOG did not inhibit the activation of ENs upon LIFU stimulation. Moreover, SST-INs activated by MG-SOG were unable to suppress the positive effect of LIFU stimulation in increasing the excitability of the LNN in the CA1 region. Subsequent behavioral analyses of SE model mice indicated that the activation of SST-INs by MG-SOG was unable to alleviate SE; moreover, although the frequency of sIPSCs was increased in the MG-SOG-mediated activation of the SST-INs group, this method did not effectively alleviate SE-related EI-IM in the hippocampal CA1 region. These data suggest that MG-SOG-mediated activation of SST-INs during the latency of SE may have a moderate effect on ISTs to ENs, which is insufficient for controlling SE and SE-related electrophysiological abnormalities in the CA1 region. However, MG-SOG-mediated activation of PV-INs effectively suppressed calcium signaling in ENs upon LIFU stimulation and further weakened the ability of LIFU stimulation to increase the excitability of the LNN in the CA1 region. Accordingly, MG-SOG-mediated activation of PV-INs delayed the initiation of SE and effectively alleviated SE, and subsequent electrophysiological experiments indicated that MG-SOG-mediated PV-IN activation could alleviate SE-related EI-IM in the hippocampal CA1 region. Both PV-INs and SST-INs are GABA-INs, and they target the perisomatic region and distal dendrites of ENs in the CA1 region, respectively; therefore, they inhibit ENs in distinct ways 37, 38. PV-INs preferentially regulate the generation and synchronization of EN APs, whereas SST-INs preferentially regulate dendritic electrogenesis 5, 32, 49. PV-INs, also called fast-spike GABAergic interneurons, presented high-frequency and stable APs, indicating that PV-INs have powerful inhibitory effects on the soma of ENs. Thus, PV-INs appear to have a stronger ability to inhibit EN excitability 37, 38, 50. As prolonged seizure episodes or multiple seizures, SE may need more powerful inhibitory effects on ENs from GABA-INs to suppress the generation or development of SEs. Therefore, MG-SOG-mediated activation of PV-INs might be more significant and meaningful. Thus, our data indicated that MG-SOG-mediated activation of PV-INs appeared to have a more obvious effect on promoting IST to ENs than MG-SOG-mediated activation of SST-INs; therefore, MG-SOG-mediated activation of PV-INs was more effective in decreasing the excitability of ENs and ultimately alleviating SE.

Moreover, we found that MG-SOG-mediated activation of PV-INs clearly increased the frequency and amplitude of sIPSCs from ENs. In chemical transmitting synapses (e.g., GABAergic inhibitory synapses), increased presynaptic release could enhance postsynaptic function. Thus, in GABAergic inhibitory synaptic transmission (GABA-INs as presynaptic components, ENs as postsynaptic components), MG-SOG-mediated activation of PV-INs could promote presynaptic GABA release, further promoting the binding of more GABA neurotransmitters to postsynaptic receptors. Thus, MG-SOG-mediated activation of PV-INs could not only increase the sIPSC frequency (reflecting presynaptic release) but also increase the sIPSC amplitude (reflecting postsynaptic function).

This noninvasive property is an advantage of ultrasound neurostimulation, which has great potential for regulating brain functions and treating multiple CNS diseases. In sonogenetics, AAV-mediated gene intervention is considered an effective way to improve the spatial specificity of ultrasound neurostimulation. Recently, stereotactic injection of AAV-mediated transfection of mechanosensitive ion channels into brain regions, an invasive intervention, has been frequently used in previous sonogenetics studies; we used this invasive method in our study to express MscL-G22S in target cells. Indeed, this method of stereotactic injection reduces the noninvasiveness of ultrasound neurostimulation, which is a disadvantage of sonogenetics. We noted that AAV-mediated gene intervention has several noninvasive delivery approaches, such as intravenous infusion and nasal inhalation, as demonstrated by previous studies 48, 51, 52. Recently, the efficacy and safety of intravenous infusion and nasal inhalation AAV-mediated gene intervention have been validated preliminarily in clinical studies and animal experiments 48, 51, 52. Thus, we assume that sonogenetics with AAV-mediated gene interventions via intravenous infusion or nasal inhalation delivery may maintain the noninvasive property of ultrasound stimulation or improve the invasive property of sonogenetics, making them promising candidates for sonogenetics-based neurostimulation.

## Conclusion

This study revealed the different effects of LIFU stimulation alone, MG-SOG-mediated activation of SST-INs, and MG-SOG-mediated activation of PV-INs in SE. Importantly, we found that the MG-SOG-mediated activation of PV-INs had a positive effect on relieving SE. This study has fundamental implications for basic research on sonogenetics and the design of sonogenetics techniques for alleviating SE or seizure activity and aids our understanding of the underlying mechanism of SE.

## Materials and methods

### Animals

All mouse experiments performed in this study were approved by the Institutional Animal Care and Use Committee of Chongqing Medical University, Chongqing, China. C57BL/6 wild-type, SST-cre, and PV-cre mice were bred and maintained according to the protocols provided by the Jackson Laboratory. Adult male mice (8-10 weeks old and weighing 20-25 g) were used in those experiments. All the mice were housed in a temperature-controlled room (approximately 22°C) with a 12 h light‒dark cycle and given free access to food and water.

### KA-induced SE mouse model and behavioral analysis

KA-induced seizures were classified according to Racine's scale 53. To induce SE, KA (25 mg/kg; MedChemExpress, USA) was administered i.p. after KA administration 54, and seizure activity in the mice was monitored continuously with a video recording system. GSs were defined as seizure events of stage 4 or 5 on Racine's scale 53. SE was defined as prolonged GSs persisting for more than 5 min without termination or recovery to baseline 49. After monitoring, the mice were i.p. injected with 10 mg/kg diazepam to terminate SE. The mice in the control group were i.p. injected with the same volume of 0.9% saline. The time between KA injection and the onset of GSs was defined as the latency to GSs. The time between KA injection and the onset of the first bout of SE was defined as the latency to SE. The percentage of GSs (%) was defined as the total duration of GSs relative to the total duration of behavioral monitoring.

Hippocampal LFPs were recorded to confirm the induction of KA-induced SE in the mice. The procedure for LFP recording was described previously 55. Electrodes were implanted in the hippocampal CA1 region at the following coordinates: -1.85 mm anterior-posterior (A-P), 1.42 mm medial-lateral (M-L), and -1.42 mm dorsal-ventral (D-V). A MAP data acquisition system (Plexon, USA) was used to monitor and record LFPs. LFPs were further analyzed via Neuroexplorer software (Nex Technologies, USA). Continuous polyspike discharges with a high amplitude (more than 2 times the baseline value) during SE were defined as KA-induced SE discharges; a representative image of LFP recordings from SE conditions was obtained approximately one hour after KA injection, which was considered a stable stage of SE conditions **(Figure 1A)**.

### AAV construction and stereotactic injection

For MG-SOG, we used an rAAV-9 vector expressing the human synapsin (hSyn) promoter, which preferentially transfects neurons. The MscL-G22S sequence was fused with the green fluorescent protein EGFP. A Cre-dependent AAV expressing MscL-G22S and EGFP (AAV-hSyn-DIO-MscL-G22S-EGFP) was injected into PV-cre and SST-cre mice to induce the expression of MscL-G22S-EGFP in PV-INs and SST-INs, respectively; a Cre-dependent AAV expressing EGFP without MscL-G22S (AAV-hSyn-DIO-EGFP) was used as a control. For calcium fiber photometry, we used the green calcium indicator GCaMp6s (or GCaMp6m) and the red calcium indicator jRGECO1a. AAV-hSyn-GCaMp6s was used to evaluate neuronal calcium signaling in the CA1 region. An AAV expressing the CaMK2α promoter was used to transfect ENs preferentially. Thus, both AAV-CaMK2α-GCaMp6s and AAV-CaMK2α-jRGECO1a were used to evaluate calcium signaling in ENs in the CA1 region upon exposure to LIFU stimulation. An AAV expressing the vesicular GABA transporter (VGAT) promoter was used to preferentially transfect GABA-INs. AAV-VGAT-GCaMp6m was used to evaluate calcium signaling in GABA-INs in the CA1 region. Cre-dependent AAV-hSyn-DIO-jRGECO1a was used to evaluate calcium signaling in PV-INs and SST-INs in the CA1 region in PV-cre mice and SST-cre mice, respectively. AAV-CaMK2a-mCherry was used to label ENs in the CA1 region. High-titer viruses (more than 2*10^12^) were purchased from BrainVTA (China). For stereotaxic injection, the mice were anesthetized with inhaled isoflurane (3% induction, 2% maintenance). The head of each mouse was fixed in a stereotaxic frame (RWD Life Science, China). A volume of 0.5 μl of AAV was stereotactically injected into the right hippocampal CA1 region (-1.85 mm A-P, 1.42 mm M-L, and -1.42 mm D-V) at a rate of 0.1 μl/min with a 5-μl syringe. The syringe was kept in place for an additional 5 min, after which it was withdrawn slowly to prevent reflux. After 4 weeks, the AAV transfection efficiency was determined via fluorescence staining, or other relevant experiments were performed.

### Calcium fiber photometry

Neuronal calcium signaling in the CA1 region was recorded with a fiber photometry system (Thinker Tech Nanjing Bioscience, Inc., China) 24, 56. After the injection of an AAV carrying a calcium indicator, a ceramic ferrule was implanted into the hippocampal CA1 region (-1.85 mm A-P, 1.42 mm M-L, -1.42 mm D-V) and fixed with a skull-penetrating screw (implanted around the bregma) and dental acrylic. The mice were housed individually and allowed to recover for at least one week. To detect calcium indicator fluorescence (GCaMp6s, GCaMp6m, or jRGECO1a), a 470 nm laser or a 580 nm laser (OBIS) were used, which were reflected by one dichroic mirror (Thorlabs), focused by a 10x objective lens (Olympus) and then coupled to an optical commutator (Doric Lenses)^57^. A two-meter optical fiber (200 mm O.D., NA = 0.37) transmitted and guided the light between the commutator and the implanted ceramic ferrule in the CA1 region. The laser power was adjusted to a low level (20-40 μW for the 470 nm laser; 20-30 μW for the 580 nm laser) at the tip of the optical fiber via a laser power meter (SANWA). The fluorescence signals were bandpass filtered (Thorlabs) and collected with a photomultiplier tube (Hamamatsu). An amplifier (Hamamatsu) was used to convert the photomultiplier tube current output to voltage signals, which were further filtered through a lowpass filter. The analog voltage signals were digitized at 500 Hz and recorded by Thinker Tech fiber photometry software (Thinker Tech Nanjing Bioscience, Inc., China) 24, 56. The data were further analyzed with MATLAB software (version R2017b, MathWorks, USA). The changes in fluorescence upon LIFU stimulation are shown as ΔF/F and were calculated via the following equation: (ΔF/F) = (F-F0)/F0, where F0 is the baseline fluorescence signal prior to LIFU stimulation (5 s for a single pulse of LIFU stimulation; 5-30 s for continuous pulses of LIFU stimulation). The highest value obtained upon LIFU-stim (within 20 sec after LIFU-stim) was defined as the peak ΔF/F, and these data were exported from MATLAB software. A small amount of dental acrylic was applied backward of the ceramic ferrule (toward the posterior fontanelle) to leave sufficient room for skull drilling (-3.85 mm A-P, 1.42 mm M-L) via LIFU-stim, where the tip of the ultrasound transducer coated with ultrasound gel was positioned **(Figure S1)**.

### Ultrasound stimulation *in vivo*

The mice were anesthetized with inhaled isoflurane. The heads of the mice were fixed in a stereotaxic frame and then shaved, and ultrasound gel was applied to the target region of the skull to promote acoustic coupling. To focus the acoustic field over the right hippocampus, the transducer tip was placed above the drill hole in the skull (A-P: -3.85 mm; M-L: 1.42 mm; right side), and the transducer was positioned at a sagittal angle of 50° above the skull **(Figure S1)**. Focal acoustic pressure was measured with a needle hydrophone (ONDA HNA-0400, USA). The mice were stimulated with an acoustic pressure of 0.38 MPa, a central frequency of 650 kHz and an intensity of 2 W/cm^2^ **(Figure S1)**; the duration of a single pulse of LIFU-stim was 1 second, with an interstimulation interval of 15 seconds for the application of continuous pulses of LIFU-stim. To monitor the temperature in the hippocampal CA1 region upon LIFU stimulation, a needle thermometer (Physitemp, USA) was implanted into the CA1 region (A-P: -1.85 mm, M-L: 1.42 mm, D-V: -1.42 mm)** (Figure S6)**. For LFP recording upon LIFU stimulation, an LFP electrode was implanted into the CA1 region (A-P: -1.85 mm, M-L: 1.42 mm, D-V: -1.42 mm)** (Figure S1)**. Sufficient room was left posterior to the needle thermometer or LFP electrode (toward the posterior fontanelle) for skull drilling for LIFU-stim, where the tip of the ultrasound transducer coated with ultrasound gel was positioned (A-P: -3.85 mm, M-L: 1.42 mm). The raw LFP data were bandpass filtered via NeuroExplorer software (USA). FGR and RO data were obtained by bandpass filtering at frequencies of 90-150 Hz and 110-200 Hz, respectively^32^.

### Morris water maze test

The Morris water maze test was used to evaluate hippocampus-dependent spatial learning and memory. A circular pool (diameter: 1.0 m; height: 0.5 m) containing 20-22°C water was used, and an escape platform (diameter: 10 cm) was submerged 1.0 cm below the water surface. White titanium dioxide was added to the pool to make the water opaque. The pool was divided into four quadrants (platform zone, left quadrant, right quadrant, and opposite quadrant). Four training trials per day were performed for four consecutive days, and a probe trial, in which the platform was removed, was performed 24 hours after the last training session. The mice were monitored by a camera mounted on the ceiling directly above the pool with shadowless light. All trials were recorded via TopScan software (CleverSys Inc., USA) for subsequent data analysis. The escape latencies of the mice in the training trials were recorded and analyzed. In the probe trial, the time spent in the platform quadrant (time in the target zone, seconds) was recorded and analyzed.

### Preparation of histological samples

Mouse brain tissues were fixed with 4% paraformaldehyde for 24 h, sequentially incubated with graded sucrose solutions for 24 h and sectioned into 10 μm frozen sections for immunofluorescence staining. Mouse brain tissues were fixed with 4% buffered formalin for 24 h, embedded in paraffin and sectioned at a thickness of 5 μm for H&E staining and immunohistochemical staining.

### Immunofluorescence staining

Frozen sections were air-dried at room temperature. Next, the sections were washed with phosphate-buffered saline (PBS) and permeabilized with 0.4% Triton X-100. The sections were subsequently washed with PBS and blocked with goat serum. The sections were subsequently incubated with primary antibodies overnight at 4°C. The following primary antibodies were used: rabbit c-fos antibody (Cell Signaling Technology; 1:200), rabbit CaMK2α antibody (Abcam; 1:200), guinea pig Vglut1 antibody (Millipore Sigma; 1:500), mouse GAD67 antibody (Millipore Sigma; 1:500), rabbit somatostatin antibody (ImmunoStar; 1:200), rabbit parvalbumin antibody (Abcam; 1:200), rabbit VGAT (Proteintech; 1:100), and rabbit NeuN antibody (Abcam; 1:200). The next day, after they were sufficiently washed with PBS, the sections were incubated with secondary antibodies for 60 min at 37°C in the dark. The following secondary antibodies were used: CoraLite-488-labeled goat anti-rabbit IgG (Proteintech; 1:100), Alexa Fluor 647-labeled goat anti-guinea pig IgG (Bioss; 1:500), CoraLite-594-labeled goat anti-mouse IgG (Proteintech; 1:100), Alexa Fluor 647-labeled goat anti-rabbit IgG (Bioss; 1:500), and DyLight-594-labeled goat anti-rabbit IgG (Abbkine; 1:200). Next, the sections were washed with PBS and mounted with 50% glycerol in PBS. Finally, fluorescence images of the sections were captured with a laser scanning confocal microscope (Nikon, Japan) or a fluorescence microscope (Nikon, Japan). The number of c-fos-positive cells was measured with ImageJ software.

### Immunohistochemical staining and HE staining

Mouse brain tissues were fixed with 4% buffered formalin for 24 h, embedded in paraffin and sectioned at a thickness of 5 μm. The paraffin sections were deparaffinized in xylene, rehydrated in a graded ethanol series, and incubated with H_2_O_2_. For antigen retrieval, the sections were placed in sodium citrate buffer and then heated in a microwave oven. The sections were subsequently blocked with bovine serum albumin. The sections were incubated with primary antibodies at 4°C overnight. The following primary antibodies were used: mouse NeuN antibody (Abcam; 1:1000), rabbit GFAP antibody (Abcam; 1:1000), and goat Iba1 antibody (Abcam; 1:500). The following day, after the sections were washed with PBS, they were incubated with secondary antibodies for 60 min at 37°C and then treated with an avidin-biotin-peroxidase complex. Next, the sections were washed with PBS. Immunoreactivity was observed with 3,3'-diaminobenzidine, and counterstaining was conducted with Harris hematoxylin. Cells with brown staining were considered positively stained. For H&E staining, paraffin sections were deparaffinized and rehydrated in the same manner as described for immunohistochemical staining and then immersed in H&E staining solution (Beyotime, China). Next, the sections were washed with distilled water. Images were acquired with a light microscope (Olympus, Japan).

### Patch-clamp recording

The mice were deeply anesthetized with sodium pentobarbital (50 mg/kg; intraperitoneal injection). The brains were rapidly removed from the mice, and 300 μm thick coronal brain slices containing the hippocampus were cut with a vibratome (Leica, Germany) in ice-cold (0-4°C) cutting solution that was bubbled with carbogen continuously. Then, the fresh brain slices were transferred to an incubation chamber containing artificial cerebrospinal fluid (ACSF) and incubated at 34°C for 60 min; the chamber was also bubbled with carbogen continuously.

To record APs, 3-6 MΩ polished glass pipettes were filled with the following internal solution (in mM): 60 K_2_SO_4_, 60 NMG, 40 HEPES, 4 MgCl_2_, 0.5 BAPTA, 12 phosphocreatine, 2 Na_2_ATP, and 0.2 Na_3_GTP; pH 7.25. APs were recorded in current-clamp mode. A current-step protocol (from -50 pA to 130 pA, with a 20 pA increase; 500 ms per current injection) was used to evoke APs.

To record sEPSCs in ENs, glass pipettes were filled with the following internal mixture (in mM): 130 Cs-methanesulfonate, 10 HEPES, 10 CsCl, 4 NaCl, 1 MgCl_2_, 1 EGTA, 5 NMG, 5 MgATP, 0.5 Na_3_GTP, and 12 phosphocreatine; pH 7.25. sEPSCs were recorded in ACSF containing 100 μM PTX at a holding potential of -70 mV. To record sIPSCs in ENs, glass pipettes were filled with the following internal solution (in mM): 100 CsCl, 10 HEPES, 1 MgCl_2_, 1 EGTA, 30 NMG, 5 MgATP, 0.5 Na_3_GTP, and 12 phosphocreatine; pH 7.25. sIPSCs were recorded in ACSF supplemented with 20 μM DNQX and 50 μM D-APV at a holding potential of -70 mV. Clampfit 11.1 software (Molecular Devices, USA) and MiniAnalysis software (SynaptoSoft) were used to analyze the patch-clamp recordings. To investigate the SE-related electrophysiological properties of ENs in the CA1 region, we sacrificed the mice by cutting mouse slices 2 hours after KA injection.

### Statistical analysis

The normality and homogeneity of all the data were tested with the Kolmogorov‒Smirnov test and Levene's test, respectively. Normally distributed and homogeneous data are presented as the mean ± standard deviation (SD), comparisons between two groups were performed via unpaired Student's two-tailed *t* test, and comparisons among multiple groups were performed via one-way analysis of variance (ANOVA) followed by the Bonferroni *post hoc* test. Nonnormally distributed or nonhomogeneous data are presented as the median and range, two-group comparisons were performed with the nonparametric Mann‒Whitney test, and multiple-group comparisons were performed with the one-way nonparametric Kruskal‒Wallis ANOVA test. Comparisons of the AP number versus injected current curves, the CA1 temperature variation versus time curves, and the escape latency over four training days were performed via two-way repeated-measures (RM) analysis of variance (ANOVA) followed by the Bonferroni *post hoc* test. Intragroup analyses of the sums of the spectra of FGRs and ROs obtained from LFP recordings upon LIFU stimulation were performed via one-way RM-ANOVA followed by the Bonferroni *post hoc* test. Statistical significance was set at *P* < 0.05. SPSS 20.0 and GraphPad Prism 9.0 software were used for statistical analyses and graphing, respectively.

## Supplementary Material

Supplementary figures and tables.

## Abbreviations

AAV: adeno-associated virus
ACSF: artificial cerebrospinal fluid
AP: action potential
ASMs: antiseizure medications
BZPs: benzodiazepines
CaMK2α: Ca^2+^/calmodulin-dependent protein kinase 2α
CNS: central nervous system
DG: dentate gyrus
EI-IM: excitation-inhibition imbalance
ENs: excitatory neurons
EST: excitatory synaptic transmission
FGRs: fast gamma oscillations
GABA-INs: GABAergic interneurons
GAD: glutamate decarboxylase
GSs: generalized seizures
HE: hematoxylin-eosin
IST: inhibitory synaptic transmission
KA: kainic acid
LFP: local field potentials
LIFU: low-intensity focused ultrasound
LNN: local neuronal network
MG-SOG: MscL-G22S-mediated sonogenetics
PBS: phosphate-buffered saline
PV: parvalbumin
PV-INs: PV interneurons
RM-ANOVA: repeated-measures-analysis of variance
RMP: resting membrane potential
ROs: ripple oscillations
RSE: refractory status epilepticus
SD: standard deviation
SEM: standard error of the mean
sEPSCs: spontaneous excitatory postsynaptic currents
sIPSCs: spontaneous inhibitory postsynaptic currents
SST: somatostatin
SST-INs: SST interneurons
SE: status epilepticus
VGAT: vesicular GABA transporter
