# Supplementary material for: Targeted sonogenetic modulation of GABAergic interneurons in the hippocampal CA1 region in status epilepticus
Source: Theranostics. 2024 Oct 7;14(16):6373–91. doi: 10.7150/thno.96598 (PMC11488105; doi:10.7150/thno.96598)
Supplement: Supplementary file 1 — Supplementary figures and tables. [file thnov14p6373s1.pdf]

Supplementary Materials

Targeted sonogenetic modulation of GABAergic interneurons in the hippocampal CA1 region in status epilepticus

Tao Xu[1,2#], Dandan Tan[1#], You Wang[1], Chen Gong[1], Jinxian Yuan[1], Xiaolan Yang[1], Yuetao Wen[1], Yuenan Ban[1], Minxue Liang[1], Yaqin Hu[2], Yang Cao[2], Yangmei Chen[1], Haitao Ran[2]

[1]Department of Neurology, the Second Affiliated Hospital of Chongqing Medical University, Chongqing 400010, China.

[2]Department of Ultrasound, Chongqing Key Laboratory of Ultrasound Molecular Imaging, the Second Affiliated Hospital of Chongqing Medical University, Chongqing 400010, China.

Corresponding author: Haitao Ran, e-mail: ranhaitao@cqmu.edu.cn; Yangmei Chen, e-mail: chenym1997@cqmu.edu.cn.

#These authors contributed equally to this work.

This supplementary file includes

Figure S1-S9

Table S1-3

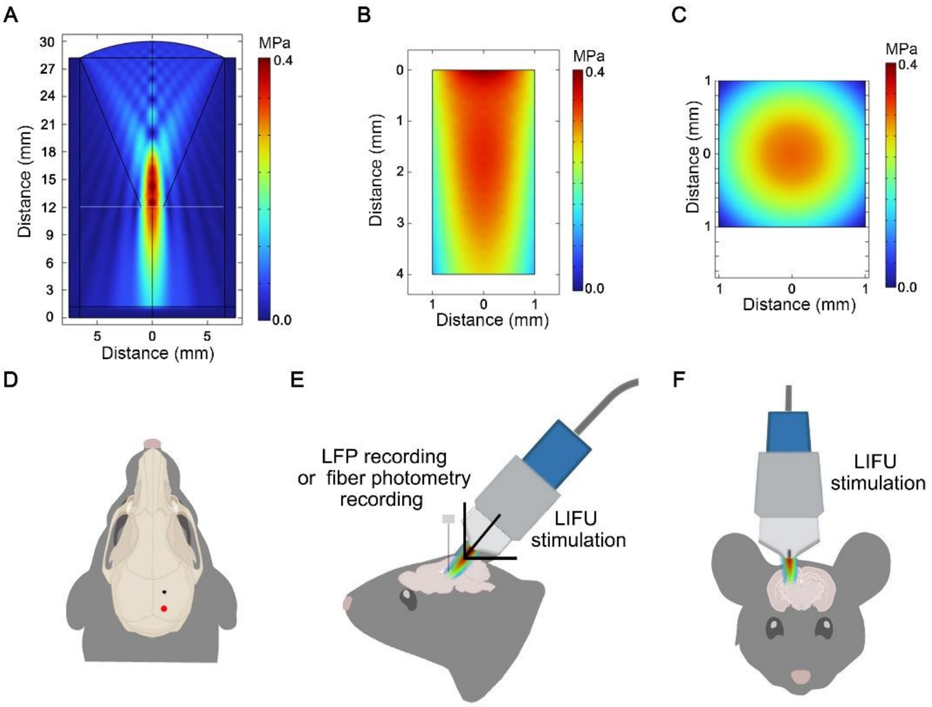

Figure S1. Illustration of the LIFU-stim setup. An ultrasound transducer was used to deliver ultrasound stimulation with a central frequency of 650 kHz and an intensity of 2 W/cm^2; the duration of each pulse of LIFU-stim was 1 s, and the interstimulation interval was 15 s for continuous pulses of LIFU-stim. A needle hydrophone (ONDA HNA-0400) was used to measure the focal acoustic pressure by repeated scanning on the X, Y, and Z axis; the acoustic pressure at the focus was 0.38 MPa. **(A)** Acoustic field in the XY plane measured in longitudinal and wide-angle views. Focal acoustic field output from the tip of the ultrasound transducer probe measured in the **(B)** XY plane and **(C)** XZ plane in cross-sectional views, respectively. **(D)** At the site on the mouse skull (A-P: -1.85 mm, M-L: 1.42 mm) indicated by the black dot, a LFP recording electrode or an optical fiber was implanted into hippocampal CA1 region to monitor LFPs and calcium signals in the CA1 region (D-V: -1.42 mm); the site on the mouse skull (A-P: -3.85 mm, M-L: 1.42 mm) indicated by the red dot was the location of skull drilling for LIFU-stim and the location where the ultrasound gel-coated ultrasound transducer tip was positioned. **(E)** Sagittal image of the mouse brain showing the ultrasound transducer at an angle of 50°. **(F)** Coronal image of the mouse brain showing the ultrasound transducer. Acoustic pressure field maps were

acquired using COMSOL Multiphysics 6.0 software.

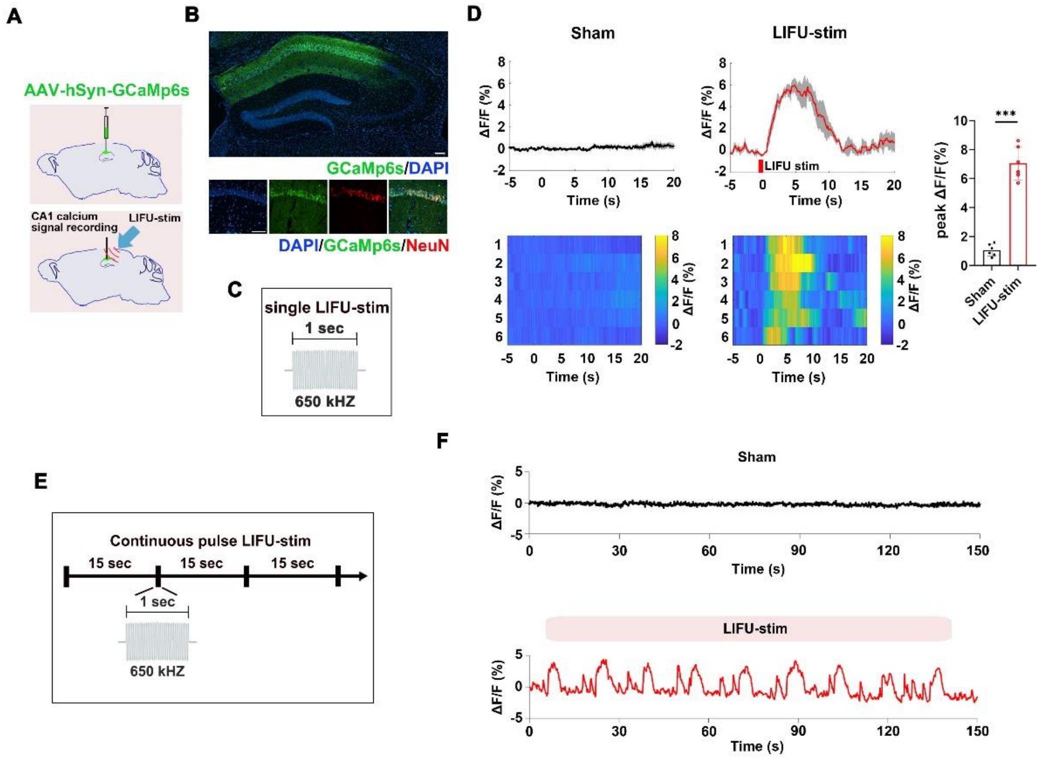

Figure S2. Neuronal calcium signals in the hippocampal CA1 region upon LIFU-stim. **(A)** Schematic diagram of stereotactic injection of an AAV expressing the hSyn promoter and the calcium indicator GCaMp6s into the hippocampal CA1 region, followed by the implantation of an optical fiber into the CA1 region for fiber photometry monitoring of neuronal calcium signals in this region upon LIFU-stim. **(B)** Representative fluorescence image of GCaMp6s in the hippocampus (*scale bar*, 100 μm) and the colocalization of GCaMp6s and NeuN, a neuronal marker, in the CA1 region (*scale bar*, 100 μm). **(C)** Schematic of a single pulse of LIFU-stim (central frequency: 650 kHz; pulse duration: 1 sec). **(D)** Neuronal calcium signals (plots of average values and heatmaps of ΔF/F, %) in the CA1 region upon application of a single pulse of LIFU-stim and the comparison of calcium signals (peak ΔF/F, %) between the sham (no LIFU-stim) group and the LIFU-stim group (6 tests for 3 mice per group). Student's t test; the data are presented as the mean ± SD; ***P < 0.001. **(E)** Schematic of continuous pulses of LIFU-stim at 15-second

intervals. **(F)** Neuronal calcium signals (ΔF/F, %) in the CA1 region upon application of continuous pulses of LIFU-stim.

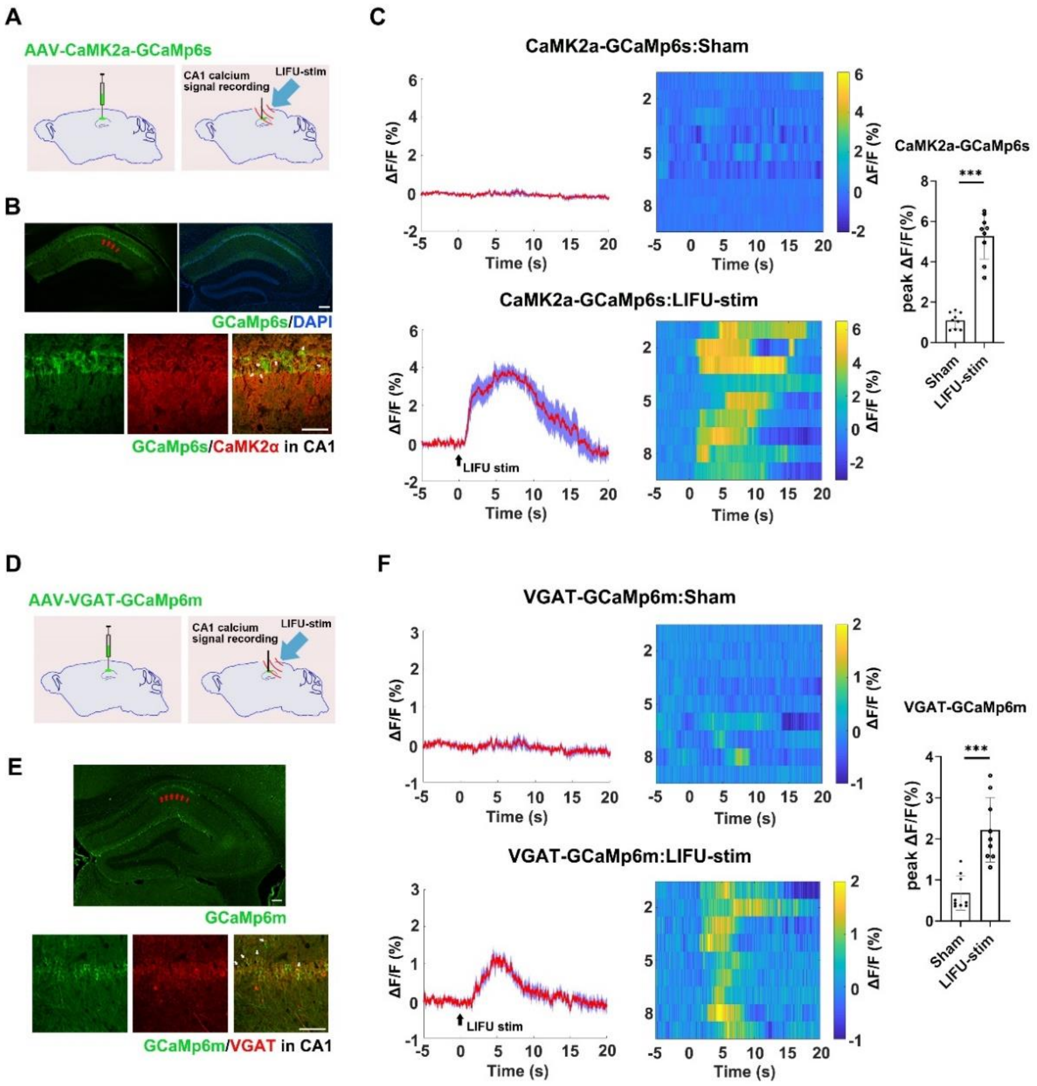

Figure S3. Calcium signals in ENs and GABA-INs in the hippocampal CA1 region upon application of a single pulse of LIFU-stim. **(A)** Schematic diagram of the stereotactic injection of AAV-CaMK2a-GCaMp6s into the hippocampal CA1 region to preferentially transfect ENs with GCaMp6s, followed by fiber photometry monitoring of neuronal calcium signals in the CA1 region upon LIFU-stim. **(B)** Representative fluorescence image of GCaMp6s in the hippocampus (the red arrows indicate GCaMp6s; *scale bar*, 100 μm) and the colocalization of GCaMp6s and CaMK2a in the CA1 region (the white arrows indicate the colocalization of

GCaMp6s and CaMK2a; *scale bar*, 50 μm). **(C)** Calcium signals (plots of average values and heatmaps of ΔF/F, %) in CaMK2a-positive ENs in the CA1 region upon application of a single pulse of LIFU-stim and the comparison of calcium signals (peak ΔF/F, %) between the sham (no LIFU-stim) group and the LIFU-stim group (9 tests for 3 mice per group). Student's *t* test; the data are presented as the mean ± SD; $^{***}P < 0.001$. **(D)** Schematic diagram of stereotactic injection of AAV-VGAT-GCaMp6m into the hippocampal CA1 region to preferentially transfect GABA-INs with GCaMp6m, followed by fiber photometry monitoring of the neuronal calcium signal in the CA1 region upon LIFU-stim. **(E)** Representative fluorescence image of GCaMp6m in the hippocampus (the red arrows indicate GCaMp6m; *scale bar*, 100 μm) and the colocalization of GCaMp6m and VGAT in the CA1 region (the white arrows indicate colocalization of GCaMp6m and VGAT; *scale bar*, 50 μm). **(F)** Calcium signal (plots of average values and heatmaps of ΔF/F, %) in VGAT-positive GABA-INs in the CA1 region upon application of a single pulse of LIFU-stim and the comparison of calcium signals (peak ΔF/F, %) between the sham group and the LIFU-stim group (9 tests for 3 mice per group). Student's *t* test; the data are presented as the means ± SDs; $^{***}P < 0.001$.

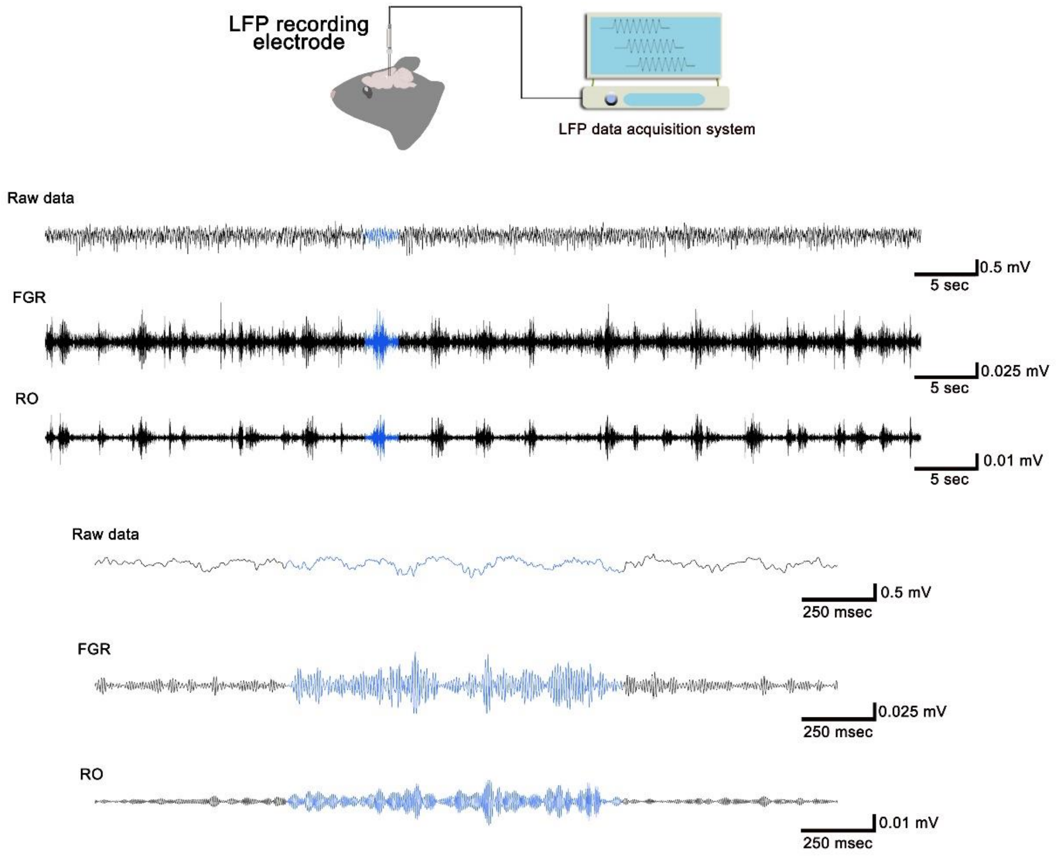

Figure S4. Schematic diagram and representative image of LFP recordings in the hippocampal CA1 region. The raw LFP data were bandpass filtered using NeuroExplorer software. FGR data were obtained by bandpass filtering at 90-150 Hz, and RO data were obtained by bandpass filtering at 110-200 Hz.

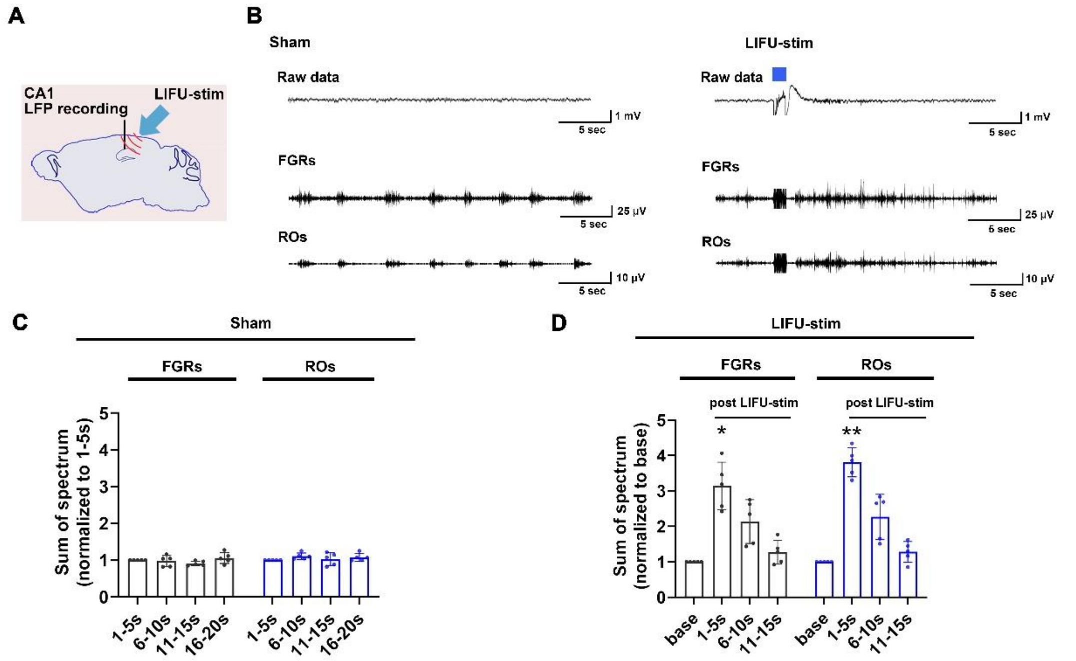

Figure S5. The effect of LIFU-stim on LFPs in the hippocampal CA1 region. **(A)** Schematic diagram of LFP recording in the hippocampal CA1 region upon LIFU-stim. **(B)** Representative traces, including raw data and FGRs and ROs, of LFPs in the hippocampal CA1 region in the sham (no LIFU-stim) group and LIFU-stim group. **(C)** The sums of spectra of FGRs and ROs (values measured 6-10 sec, 11-15 sec, and 16-20 sec were normalized to those measured 1-5 sec (base)) in the sham group (n = 5). One-way RM-ANOVA followed by Bonferroni *post hoc* test; the data are presented as the mean ± SD. **(D)** The sums of spectra of FGRs and ROs (values measured 1-5 sec, 6-10 sec, and 11-15 sec after LIFU-stim were normalized to baseline values) in the LIFU-stim group (n = 5). One-way RM-ANOVA followed by Bonferroni *post hoc* test; $*P < 0.05$ and $**P < 0.01$, 1-5 sec after LIFU-stim compared to baseline.

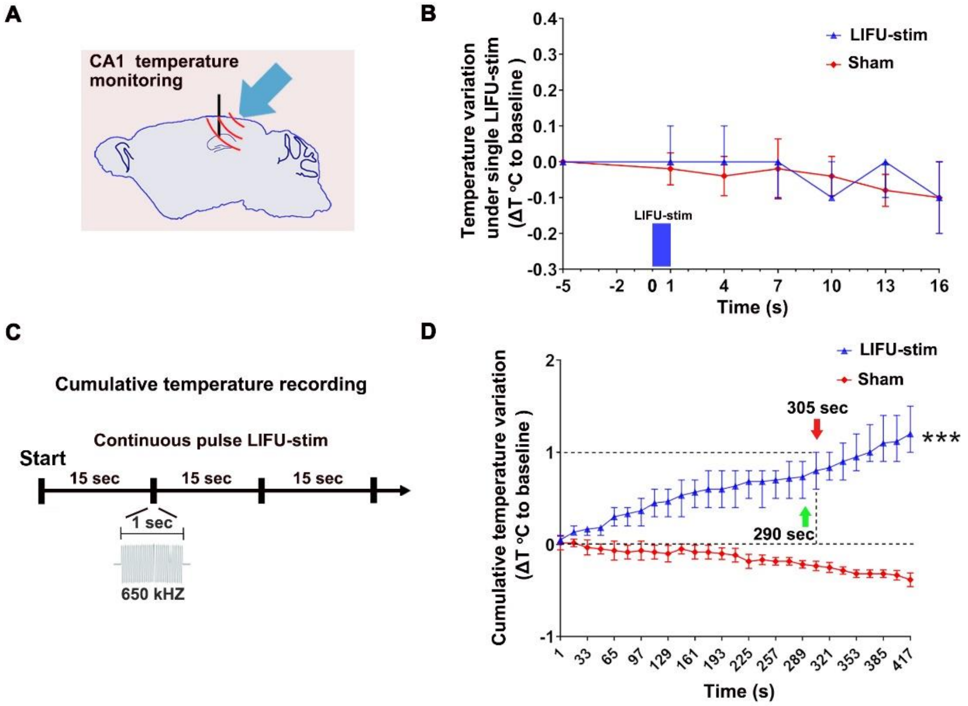

Figure S6. The effect of LIFU-stim on the temperature variation in the hippocampal CA1 region. **(A)** Schematic of temperature monitoring in the hippocampal CA1 region upon LIFU-stim. **(B)** The temperature variation (ΔT °C relative to baseline, i.e., 5 sec before LIFU-stim) in the hippocampal CA1 region upon application of a single pulse of LIFU-stim in the sham (no LIFU-stim) group and LIFU-stim group (n = 5). Two-way RM-ANOVA followed by Bonferroni *post hoc* correction; the data are presented as the mean ± range. **(C)** Schematic of cumulative temperature recording in the hippocampal CA1 region upon application of continuous pulses of LIFU-stim. **(D)** The cumulative temperature variation (ΔT °C relative to baseline, i.e., 5 sec before LIFU-stim) in hippocampal CA1 region upon application of continuous pulses of LIFU-stim in the sham group and LIFU-stim group (n = 6). At 305 seconds, indicated by the red arrow, the maximum temperature variation in the hippocampal CA1 region reached 1°C; thus, to minimize thermal effect caused by LIFU-stim, the duration of continuous pulses of LIFU-stim at an interval of 15 s was set to 290 seconds, which is indicated by the green arrow. Two-way RM-ANOVA by Bonferroni *post hoc* correction; the data are the mean ± range; ***$P < 0.001$.

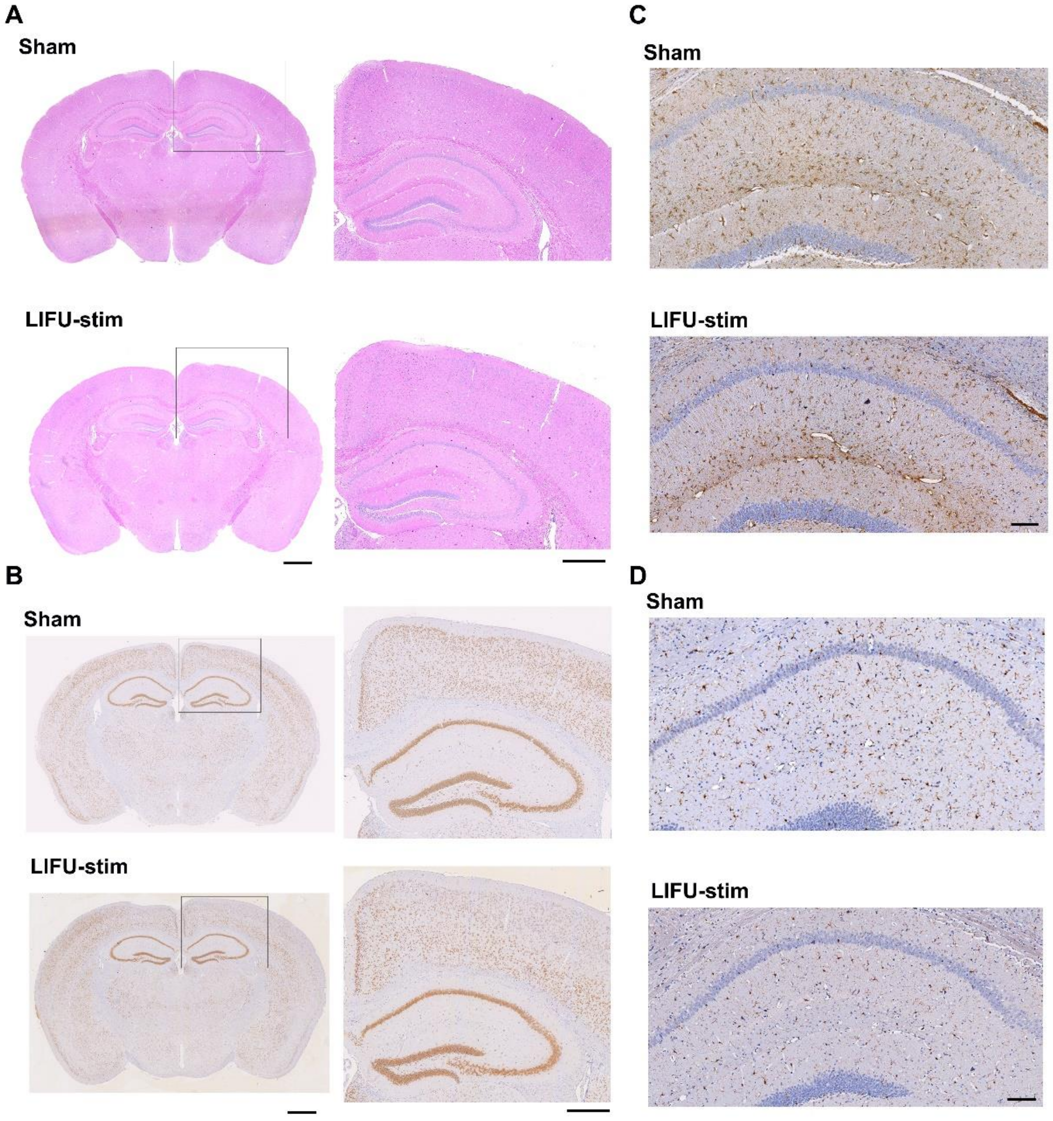

Figure S7. The effects of 290 sec of continuous pulses of LIFU-stim on mouse brain morphology. **(A)** Hematoxylin-eosin staining of mouse brains and **(B)** immunohistochemical staining of NeuN (neuronal marker) (left images enlarged from black boxes in right images; right *scale bar*, 1000 μm; left *scale bar*, 500 μm), and immunohistochemical staining of **(C)** GFAP (astrocyte marker), and **(D)** Iba1 (microglial marker) in the brains and hippocampal CA1 region (*scale bar*, 100 μm) of mice in the sham (no LIFU-stim) group and the LIFU-stim group.

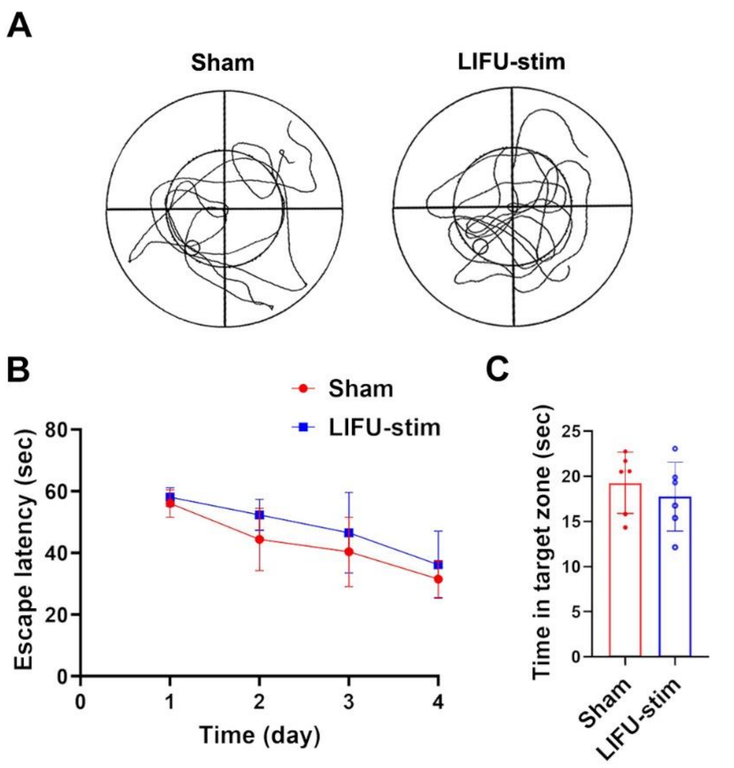

Figure S8. The effects of 290 sec of continuous pulses of LIFU-stim on cognitive function in mice. The Morris water maze test was used for evaluating cognitive function. **(A)** Representative trajectoris of mice in the sham group and the LIFU-stim group. **(B)** Escape latency (sec) over the four training days in the sham (no LIFU-stim) group and LIFU-stim group (n = 6); two-way RM-ANOVA followed by Bonferroni *post hoc* correction; the data are presented as the mean ± SD. **(C)** Comparison of the time spent in the target zone between the sham group and the LIFU-stim group (n = 6); student's *t* test; the data are presented as the mean ± SD.

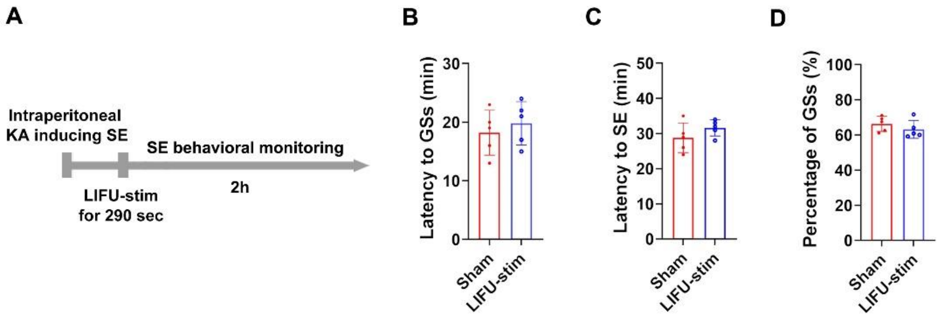

Figure S9. The effects of LIFU-stim alone on seizure events in KA-induced SE

model mice. **(A)** Schematic diagram of the experimental design; SE was induced with KA, and then continuous pulses of LIFU-stim were applied in the latency of GSs. **(B)** Comparisons of the latency to GSs (min), **(C)** latency to SE (min), and **(D)** percentage of GSs (%) between the sham group (no LIFU-stim) and the LIFU-stim group (n = 5). Student's *t* test; the data are presented as the mean ± SD.

Table S1. Intrinsic physiological properties of hippocampal CA1 CaMK2a+ cells (ENs) in Control group and SE group.

	**Con**		**SE**		
**Parameter**	**Mean**	**± SD**	**Mean**	**± SD**	***P* value**
Resting membrane potential (mV)	-67.57	2.20	-68.18	2.42	0.52
Input resistance (mOhm)	235.91	42.66	218.62	59.59	0.42
Membrane capacitance (pF)	114.06	41.21	127.34	44.75	0.46
Action potential threshold (mV)	-40.59	4.02	-39.94	6.40	0.77
Action potential half-width (ms)	3.73	0.34	3.20	0.21	< 0.01
Peak amplitude (mV)	114.75	2.64	112.93	5.18	0.29

SD, standard deviation; SE, status epilepticus; EN, excitatory neuron.

*Statistical significance was determined by Student's *t* test.

Table S2. Intrinsic physiological properties of hippocampal CA1 CaMK2a+ cells (ENs) in LIFU/EGFP group, MscL-G22S group, and LIFU/MscL-G22S group in SST-cre mice.

	**LIFU/EGFP**		**MscL-G22S**		**LIFU/MscL-G22S**		
**Parameter**	**Mean**	**± SD**	**Mean**	**± SD**	**Mean**	**± SD**	***P* value***
Resting membrane potential (mV)	-66.69	3.69	-65.98	3.49	-66.14	2.44	0.88
Input resistance (mOhm)	383.64	275.81	344.80	145.55	288.61	206.96	0.62
Membrane capacitance (pF)	107.22	30.80	93.49	46.62	103.35	34.53	0.71
Action potential threshold (mV)	-44.49	17.60	-47.38	17.73	-44.55	17.37	0.92
Action potential half-width (ms)	3.47	0.39	3.54	0.42	3.49	0.36	0.93
Peak amplitude (mV)	108.39	5.43	106.55	6.45	107.83	7.51	0.81

SD, standard deviation; EN, excitatory neuron.

*Statistical significance was determined by one-way ANOVA followed by Bonferroni *post hoc* test.

Table S3. Intrinsic physiological properties of hippocampal CA1 CaMK2a+ cells (ENs) in LIFU/EGFP group, MscL-G22S group, and LIFU/MscL-G22S group in PV-cre mice.

	**LIFU/EGFP**		**MscL-G22S**		**LIFU/MscL-G22S**		
**Parameter**	**Mean**	**± SD**	**Mean**	**± SD**	**Mean**	**± SD**	***P* value***
Resting membrane potential (mV)	-67.56	1.82	-69.10	3.00	-64.05	3.52	0.58
Input resistance (mOhm)	322.98	172.57	294.03	142.95	256.03	194.21	0.68
Membrane capacitance (pF)	114.84	39.15	112.71	60.66	106.28	30.09	0.91
Action potential threshold (mV)	-47.29	14.64	-49.68	12.35	-42.61	3.61	0.37
Action potential half-width (ms)	3.34	0.15	3.39	0.59	3.99	0.47	< 0.01
Peak amplitude (mV)	109.22	7.58	108.70	10.07	110.49	14.95	0.94

SD, standard deviation; EN, excitatory neuron.

*Statistical significance was determined by one-way ANOVA followed by Bonferroni *post hoc* test.
